# Leveraging Multi-Model Machine Learning Algorithms for Tumor–Normal Classification and Discovery of Biomarkers in Colorectal Cancer Using Multi-Omics Data

**DOI:** 10.3390/cancers18101503

**Published:** 2026-05-07

**Authors:** Duaa Mohammad Alawad, Mark Fertel, Chindo Hicks

**Affiliations:** Department of Genetics and the Bioinformatics and Computational Medicine Program, School of Medicine, Louisiana State University Health Sciences Center, 533 Bolivar Street, New Orleans, LA 70112, USA; dalaw2@lsuhsc.edu (D.M.A.); mferte@lsuhsc.edu (M.F.)

**Keywords:** gene expression, somatic mutations, machine learning, classification, colorectal cancer biomarkers

## Abstract

**Background**: Despite remarkable progress in clinical management and screening, colorectal cancer (CRC) remains a major cause of cancer-related deaths worldwide. Sadly, both the number of CRC incidences and the mortality rate are trending upwards, particularly in younger individuals. There is an urgent need for the identification of reliable diagnostic biomarkers and therapeutic targets, and the development of accurate algorithms to guide therapeutic decision-making at the point of care. Here, we leverage multi-model integrative Machine Learning (ML) algorithms using RNA-Seq and somatic mutation data for the classification of tumor–normal samples and the discovery of potential biomarkers and therapeutic targets. **Methods**: We used RNA sequencing (RNA-Seq) and somatic mutation data from The Cancer Genome Atlas (TCGA) for the development of classification models and the discovery of biomarkers and therapeutic targets. The models were validated using two independent datasets. **Results**: ML algorithms accurately classified tumor samples and identified a signature for 58 genes, which could serve as potential diagnostic biomarkers. Functional analysis revealed the Wnt and GPCR signaling pathways enriched for somatic mutations. **Conclusions**: Multi-model integrative ML algorithms integrating gene expression with somatic mutation data represent a powerful approach to the classification of tumor samples and the discovery of biomarkers.

## Simple Summary

Colorectal cancer is the second leading cause of cancer-related deaths worldwide. A critical unmet medical need in clinical management of colorectal cancer centers around the discovery of biomarkers, therapeutic targets, predictors of survival outcome, and the development of more accurate algorithms to identify individuals at high risk of developing aggressive disease, who could be prioritized for treatment. With the availability of multi-omics data, we are now well-positioned to address this critical unmet medical need. Here, we leveraged multi-model integrative Machine Learning algorithms using RNA-Seq and somatic mutation data for tumor–normal classification and the identification of clinically relevant diagnostic biomarkers and molecular targets. Machine Learning algorithms accurately classified tumor–normal samples and identified a signature for potential clinically actionable diagnostic biomarkers, therapeutic targets, and predictors of survival outcome.

## 1. Introduction

Colorectal cancer (CRC) is the third most commonly diagnosed malignancy and the second leading cause of cancer-related deaths worldwide [1]. According to the Global Cancer Observatory (GLOBOCAN)’s 2022 estimates, there were approximately 1,930,000 new cases of CRC and over 904,000 deaths worldwide [1]. The United States (US) is not spared from the burden of CRC. A recent report on cancer statistics from the American Cancer Society showed that there were 154,270 new cases of CRC in the US and 52,900 deaths from the disease in 2025 [2]. Sadly, in addition to these alarming numbers regarding the incidence and mortality rate of CRC primarily among individuals aged 50 and above [3], there is growing concern about the increasing CRC incidences and deaths among younger individuals [2]. Both recent and future projections indicate that the burden of CRC is increasing, with an estimated 3.2 million new cases and 1.6 million deaths occurring annually worldwide by 2040 [2]. This is particularly concerning because CRC has very limited treatment options, and current screening protocols, such as colonoscopy, which is usually recommended to start at age 50, are less effective in the younger population. Another major concern is that a significant proportion of CRCs are diagnosed at late stages, which drastically increases the risk of recurrence and distant metastases, and significantly reduces the survival rate [4,5]. These alarming statistics, coupled with increasing incidence and mortality rates in younger individuals, show the urgent need for the discovery of clinically actionable diagnostic biomarkers for the early detection of the disease and targets for the development of novel therapeutics. In addition, there is a critical need to develop accurate algorithms to identify individuals at high risk of developing aggressive disease, who could be prioritized for treatment to improve survival outcomes.

Much progress has been made in the clinical management of CRC. Current diagnostic procedures for CRC encompass colonoscopy, fecal occult blood testing (FOBT), blood assays for indicators such as carcinoembryonic antigen (CEA), and imaging modalities, including CT scans [6,7]. While these widely used protocols have been relatively effective, they have limitations [5]. Colonoscopy is invasive and expensive, requiring substantial patient preparation and compliance [6]. In addition, colonoscopy is generally recommended for individuals age 50 and above, and therefore is less effective for younger individuals, in whom incidences and deaths are increasing [8]. FOBT can produce false positives or false negatives. Blood-based biomarkers often lack specificity [1], while imaging may not reliably distinguish between benign and malignant lesions [9,10]. To overcome the limitations of these approaches, CRC diagnosis typically relies on a synthesis of clinical assessment, colonoscopy, and verification via tissue biopsy [11,12]. There is an urgent need to develop novel, non-invasive, accurate tools for the early diagnosis of CRC, and accurate algorithms to identify individuals at high risk of developing aggressive disease who could be prioritized for treatment to improve survival outcomes.

Recent advances in next-generation sequencing have revolutionized genomic research on CRC, a disease characterized by the progressive accumulation of somatic mutations, genomic instability, and the dysregulation of key signaling pathways, including Wnt/β-catenin signaling, extracellular matrix (ECM) remodeling, and metabolic reprogramming. Large-scale cancer genome sequencing projects, such as The Cancer Genome Atlas (TCGA), have produced large, multi-dimensional omics datasets that have enabled detailed analysis of the CRC genome, revealing the disease heterogeneity and increasing our understanding of its molecular taxonomy [13]. However, despite extensive knowledge of colorectal cancer pathogenesis, including mutation-driven tumor initiation and progression and tumor microenvironment interactions, multi-omics data have not been fully leveraged and integrated using multi-model Machine Learning (ML) algorithms for the discovery of potential clinically biomarkers, therapeutic targets, predictors of survival outcomes, and accurate tumor–normal classification to identify individuals at risk of developing aggressive disease who could be prioritized for treatment to improve clinical outcomes.

Recent advances in ML and Artificial Intelligence (AI) provide unprecedented opportunities for the development of accurate algorithms for the classification of tumors and normal samples and the discovery of biomarkers, therapeutic targets, and predictors of survival outcomes [13,14,15,16,17]. ML has been deployed in CRC research for the discovery of novel diagnostic biomarkers and in predicting treatment outcomes [16,17,18]. Previous studies have demonstrated that ML models such as Random Forest and Support Vector Machine can accurately distinguish tumors from normal samples and identify clinically relevant biomarkers in CRC [13,16,17,18,19]. Additionally, integrative multi-omics approaches combining gene expression and somatic mutation data have been shown to improve predictive performance and enhance biological interpretability [16,17,20,21,22]. These findings highlight the effectiveness of ML-based approaches in uncovering complex molecular patterns in CRC and support their growing role in precision oncology. Furthermore, clinical studies have demonstrated that ML models incorporating chromosomal instability, mutational status, and gene expression can predict chemotherapy response in patients with metastatic CRC [11]. However, to date, there is limited information on the application of multi-model integrative ML frameworks that simultaneously leverage gene expression and somatic mutation data for tumor–normal classification and the identification of clinically actionable biomarkers, therapeutic targets, and predictors of survival outcomes in CRC. Therefore, the objectives of this study were two-fold: (1) to develop and internally and externally validate a multi-model integrative ML framework incorporating multiple ML algorithms for accurate classification of tumor and normal samples, and (2) to leverage this framework to identify diagnostically relevant biomarkers and molecular targets, as well as features associated with clinical outcomes, using publicly available RNA-Seq and somatic mutation data.

## 2. Materials and Methods

### 2.1. Project Design and Execution Strategy

**Figure 1:**
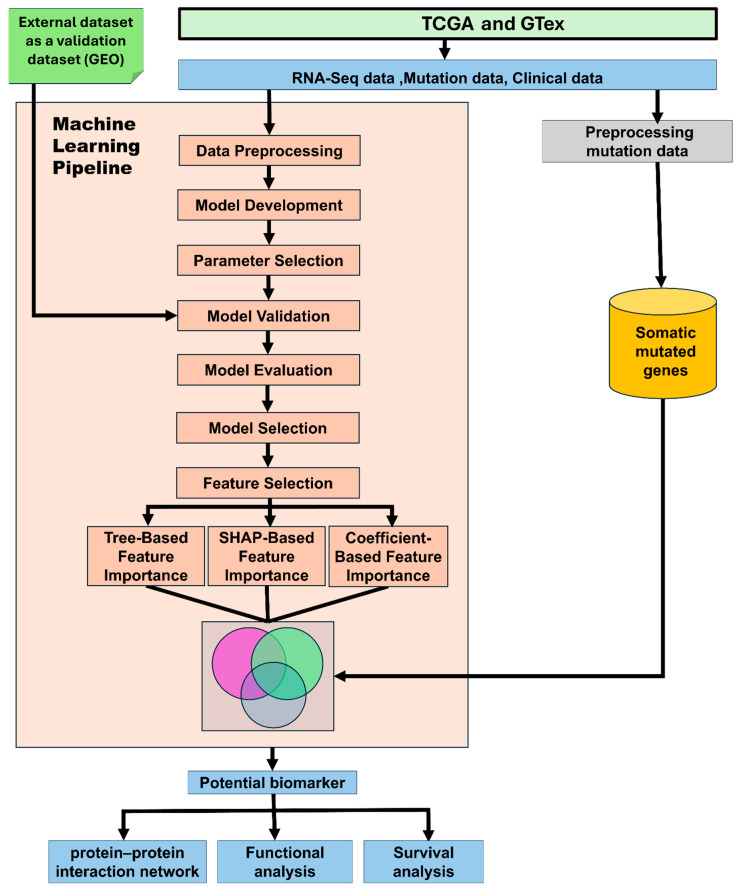
Schematic representation of the study design and integrated analytical workflow. This figure illustrates the data sources, including The Cancer Genome Atlas (TCGA) and Gene Expression Omnibus (GEO), followed by data preprocessing, model development, and multi-method feature selection. It further highlights the integration of gene expression with somatic mutation data and downstream analyses, including protein–protein interaction (PPI) network modeling, functional enrichment, and survival analysis.

The primary objective of this study was to leverage a multi-model integrative machine learning framework that utilizes gene expression and somatic mutation data for accurate tumor classification and the identification of clinically relevant biomarkers, molecular targets, and features associated with disease outcomes. The overall study design and execution workflow are presented in Figure 1. The study was conducted in five phases, which included: (1) model training and internal validation using TCGA data for accurate tumor–normal classification; (2) external validation using independent GEO datasets to assess the generalizability of the developed algorithms; (3) multi-method feature/gene importance analysis for the discovery of potential diagnostic biomarkers and therapeutic targets; and (4) modeling a protein–protein interactions network and conducting a functional enrichment analysis, integrating somatic mutation information and using Gene Ontology (GO) analysis to characterize the molecular functions, biological processes, and cellular components in which ML-identified genes are involved; and (5) survival analysis to test the ability of ML-identified genes to predict survival outcomes. Details about all the analytical steps are described in the subsections below. Additional information, including details about statistical computing for each step, is provided in the Appendix A.

### 2.2. Sources of RNA-Seq, Somatic Mutation, and Clinical Data

We used publicly available RNA-Seq and somatic mutation datasets from the same individuals in The Cancer Genome Atlas to design, train, test, and internally validate the machine learning models, as well as to identify clinically relevant biomarkers and molecular targets associated with disease outcomes. The developed ML models were externally validated using two independent datasets from the Gene Expression Omnibus (GEO) [14,15] to assess the robustness and generalizability of the developed ML algorithms across heterogeneous datasets.

RNA-Seq, somatic mutation, and corresponding clinical metadata were separately downloaded from the Genomics Data Commons (GDC) portal (https://gdc.cancer.gov/) using the GDC Data Transfer Tool (v2.3) [11] (accessed on 29 September 2025). RNA-Seq data comprised raw gene-level read counts for tumors and normal samples, providing comprehensive transcriptome-wide expression profiles. After data were downloaded, individual sample files were consolidated into a unified gene expression data matrix consisting of a total of n = 1043 samples distributed as n = 989 tumor samples and n = 54 normal/control samples containing 60,660 transcripts/genes. Using sample identifiers, we linked molecular data with clinical information, which included survival status (alive or dead), and retained only those samples that contained complete information across the two data types.

Due to the unbalanced study design in terms of the distribution of samples, which can result in sampling errors, we sought to increase the number of control samples to create a balanced study design. To address this need, we downloaded additional n = 822 normal/control colon samples from the Genotype-Tissue Expression (GTEx) project [22]. The GTEx data contained 57,562 transcripts/genes. We then merged the TCGA with GTEx normal samples, creating a final merged cohort comprising n = 989 tumor and n = 876 normal/control samples used for model development and internal validation. After merging the two datasets, we processed the data to retain only common genes found in both datasets. This processing resulted in a total of 57,560 transcripts/genes in the merged dataset used in the study.

Somatic mutation data from TCGA were generated using the whole-exome sequencing of tumor samples and were downloaded from the GDC portal using the data Transfer Tool (v2.3) [11] in Mutation Annotation Format (MAF) file. Somatic mutation data were generated using the same tumor samples used to generate the RNA-Seq data, consistent with the TCGA protocol. This mutation file included information on mutation types, somatic mutated genes, chromosome positions, mutation locations, and the corresponding samples in which mutations were detected. The mutations file included 20,303 somatic mutated genes containing single-nucleotide polymorphisms (SNPs), insertions (INSs), and deletions (DELs) [23]. Using sample identifiers, we linked somatic mutation data with clinical information to retain only those samples that contained complete information across the two data types. We annotated and processed somatic mutation data using the Maftools R package (version 2.24.0), a specialized tool for analyzing and visualizing mutation annotation files [24]. The original somatic mutation data file is available for download from the GDC: https://gdc.cancer.gov (accessed on 29 September 2025). The processed somatic mutation data files were assembled in a mutation data matrix used in downstream integrative analysis and to quantify the number of somatic mutations per gene. The mutations were characterized as missense, nonsense, frameshift, and splice site. This information was used to assess whether the mutations were pathogenic and whether the genes they map to are driver genes. A gene was considered a driver if it contained driver mutations.

The development and application of ML algorithms require that the developed methods are validated on an independent dataset to ensure the rigor, reproducibility, and generalizability of the developed methods. To address these requirements and evaluate the generalizability and robustness of the developed ML models, we used an external independent validation dataset derived from two independent GEO datasets (GSE50760 and GSE251845) [14,15]. The independent dataset comprised a total of n = 98 samples (n = 58 tumors and n = 40 controls/normal), selected to provide variability in patient demographics and experimental conditions. These external datasets were used exclusively for independent validation, without any additional model training or parameter tuning, to provide an unbiased assessment of model performance. Table 1 shows the sources, types, and distribution of the datasets used in this study.

### 2.3. Machine Learning Pipeline

The machine learning (ML) pipeline implemented in this study was designed as a structured, end-to-end analytical framework to ensure robust, reproducible, and generalizable tumor–normal classification and biomarker discovery. The pipeline integrated multiple sequential stages, including data preprocessing and quality control, feature harmonization, model development, internal cross-validation, external validation, and multi-method feature selection. High-dimensional RNA-Seq data were processed and normalized to minimize technical variability and ensure consistency across datasets, followed by feature alignment to retain shared genes across TCGA, GTEx, and GEO cohorts. A diverse set of supervised ML algorithms, encompassing linear, kernel-based, and ensemble learning methods, was employed to capture complementary patterns within the data and reduce model-specific bias. Model performance was rigorously evaluated using stratified 10-fold cross-validation and independent external validation to assess generalizability across heterogeneous datasets. To further enhance the biological interpretability and robustness of biomarker discovery, feature importance was quantified using three complementary strategies: tree-based, SHAP-based, and coefficient-based methods. This integrated pipeline enabled not only the accurate classification of tumor and normal samples but also the identification of stable and reproducible gene signatures associated with colorectal cancer.

#### 2.3.1. Data Processing and Quality Control

The processing and quality control of the datasets were critical steps in developing and validating generalizable machine learning models for tumor–normal classification in colorectal cancer (CRC), as well as for identifying diagnostically relevant biomarkers and molecular targets associated with clinical outcomes. To ensure optimal performance and reproducibility, we implemented a systematic data processing pipeline in Python (v3.10.3), utilizing key modules such as Pandas (v2.2.2) [25], Scikit-learn (v1.4.2) [26], and Imblearn (v0.12.3) [27]. The pipeline consisted of sequential preprocessing steps, including data harmonization, clinical metadata annotation, label encoding quality filtering, denoising, log-transformation, normalization, sample shuffling, and sample class balancing to eliminate any sampling errors. Each step was designed and carried out to minimize technical biases and to enhance downstream analytical accuracy.

RNA-Seq used for training and testing from TCGA and the GEO datasets used exclusively for external validation were processed independently using the harmonized pipeline. The TCGA expression dataset initially contained 60,660 transcripts. Transcripts with very low expression values (i.e., genes with near-zero counts across samples) were filtered out to reduce noise by removing non-informative genes. The remaining expression values were normalized and corrected for sequencing depth and library size differences. The resulting data were log_2_-transformed to stabilize variance and mitigate scale-related effects. After these preprocessing steps, the data expression matrix retained 26,348 high-confidence transcripts. Probes represented by Ensemble identifiers were then mapped to gene symbols, yielding a final set of 26,348 expressed genes used for the development of ML algorithms. RNA-Seq and mutation datasets were preprocessed separately and subsequently annotated with clinical metadata prior to integration into the analytical framework, as described in the subsection on data integration.

For external validation, two independent RNA-Seq datasets with GEO accession numbers GSE50760 [13] and GSE251845 [14] were processed. Both datasets were generated using Illumina sequencing platforms and processed using the same standardized workflow applied to TCGA, as described in the preceding paragraph. After preprocessing and annotation, the combined GEO dataset comprised 13,711 expressed genes. To ensure consistency among input features across TCGA, GTEx, and GEO datasets, we retained the 13,469 genes common to all three datasets. This intersection step preserved feature alignment, minimized batch effects, and maintained comparability across model training, testing, internal, and external validation without merging datasets.

To prevent ordering bias during model training, TCGA samples were randomly shuffled using a fixed random seed. Binary class labels (“tumor” and “normal”) were numerically encoded using the LabelEncoder from Scikit-learn (v1.4.2) to facilitate binary classification. Subsequently, Z-score normalization was applied using StandardScaler (v1.4.2), adjusting each gene’s expression to zero mean and unit variance. To prevent data leakage, the scaler was fitted exclusively to TCGA data and then applied to the external GEO datasets. The final preprocessed, quality-controlled, normalized, feature-aligned, and platform-harmonized datasets were used for feature selection, model construction, cross-validation, and independent external validation [28].

#### 2.3.2. Development and Implementation of ML Models

Using processed, quality-controlled, normalized data, we developed an ML pipeline and implemented it as a structured, multi-stage process encompassing model development using multiple supervised ML algorithms. Recognizing the complexity and high-dimensional nature of transcriptomic data, we incorporated a variety of ML model architectures to capture the linear decision boundaries, nonlinear interactions, and hierarchical relationships among features/genes.

We trained and evaluated seven state-of-the-art supervised ML model classifiers, which included: Random Forest (RF) [29], Extra Trees (ET) [30], Support Vector Machine (SVM) [31], Gradient Boosting (GB) [32], Extreme Gradient Boosting (XGB) [33], Light Gradient Boosting Machine (LGBM) [34], and Logistic Regression (LR) [35]. These algorithms represent a spectrum of modeling strategies, including linear models (Logistic Regression), kernel-based methods (Support Vector Machine), ensemble tree-based approaches (Random Forest, Extra Trees), and gradient-boosted learners (XGBoost (v2.0.3), LightGBM (v4.3.0), Gradient Boosting (v1.4.2)). The selection of these models was motivated by their complementary strengths in handling high-dimensional transcriptomic data. Linear models provide interpretability and robustness, kernel-based methods capture complex nonlinear decision boundaries, and ensemble and boosting approaches effectively model feature interactions, reduce variance, and improve predictive accuracy. Importantly, this multi-model strategy reduces model-specific bias and increases confidence in biologically meaningful feature selection. This diverse model selection enables a comprehensive evaluation of classification performance and ensures robust and generalizable biomarker discovery. Details about the computational aspects of each model are provided in Appendix A.1.

For model development, we performed the following steps: hyperparameter optimization, stratified internal validation, independent external validation, comprehensive model evaluation and selection. These stages were designed to ensure robust tumor–normal classification, minimize overfitting, and ensure the robustness and generalizability of the developed methods. Critical steps in model development and implementation involved an internal validation and evaluation of the developed models to rigorously assess model reliability and reduce the risk of overfitting. To address this critical need, we employed stratified 10-fold cross-validation (10-FCV) during internal model validation [36]. Using this approach, each fold maintained the same tumor-to-normal sample ratio as the original dataset, thereby preserving class distribution throughout training and testing. That is, in each iteration, 90% of the data were used to train the model, while the remaining 10% served as a test set. This process was repeated ten times, with each sample appearing once in the test set and nine times in the training set.

The resulting performance metrics were averaged across all folds to provide robust, unbiased estimates of model performance. We used stratified 10-FCV because it offers several advantages over a single train–test split. The approach provided reliability by evaluating the model multiple times across different data subsets, thereby reducing sensitivity to random partitioning. In addition, the approach helped prevent overfitting by repeatedly training and validating the model on separate subsets. Moreover, the approach maximized data usage, which is particularly important when the number of samples is limited. The 10-FCV also allowed for an examination of performance variance across folds, thereby enabling assessment of model stability and generalizability, which in turn informed model selection.

#### 2.3.3. Model Evaluation

**Figure 2:**
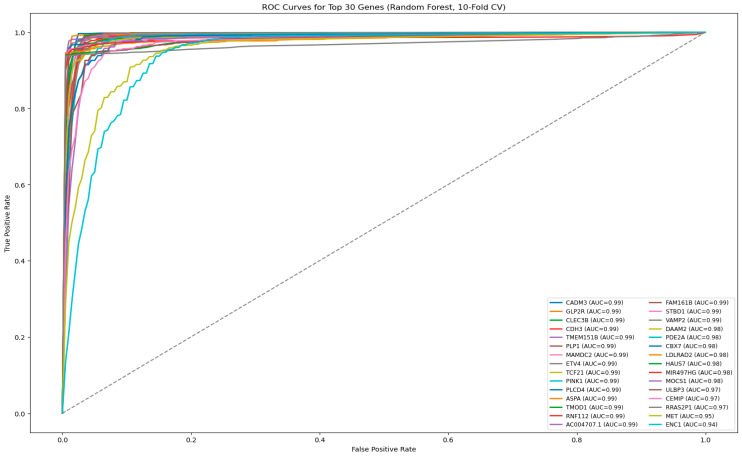
Receiver operating characteristic (ROC) curves for the top 30 genes identified through integrated tree-based feature importance analysis. Each gene was independently evaluated using a univariate Random Forest classifier with stratified 10-fold cross-validation. The x-axis represents the false positive rate, and the y-axis represents the true positive rate. Each curve corresponds to a single gene, with the area under the curve (AUC) indicating its diagnostic performance in distinguishing colorectal tumor from normal samples.

**Figure 3:**
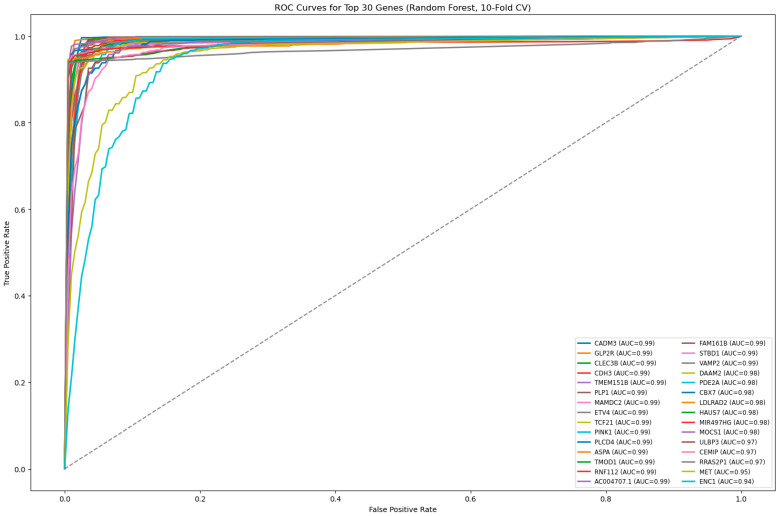
Receiver operating characteristic (ROC) curves for the top 30 genes prioritized using aggregated SHAP importance scores across five ensemble models. Each gene was independently evaluated using a univariate Random Forest classifier with stratified 10-fold cross-validation. The x-axis represents the false positive rate, and the y-axis represents the true positive rate. Each curve corresponds to a single gene, with the area under the curve (AUC) indicating its diagnostic performance in distinguishing colorectal cancer (CRC) from normal tissue.

**Figure 4:**
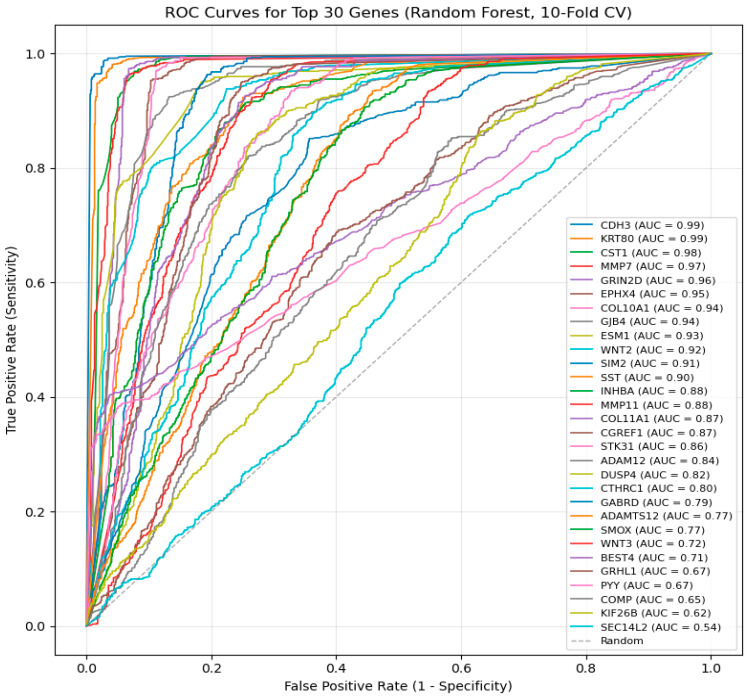
Receiver operating characteristic (ROC) curves for the top 30 genes prioritized using coefficient-based feature importance derived from linear SVM and Logistic Regression models. Each gene was independently evaluated using a univariate Random Forest classifier with stratified 10-fold cross-validation. The x-axis represents the false positive rate, and the y-axis represents the true positive rate. Each curve corresponds to a single gene, with the area under the curve (AUC) indicating its diagnostic performance in distinguishing colorectal cancer from normal samples.

Following the internal model validation phase, a final evaluation of the ML models was conducted to compare their predictive performance on an independent dataset. This phase was crucial in assessing how well each model generalizes to unseen independent data, ensuring that the selected models provide reliable and clinically relevant predictions. To evaluate each ML model, we used five classification metrics that comprehensively measure different aspects of model performance, as follows: (i) Accuracy was used as the baseline measure of the overall proportion of correctly classified samples. However, because accuracy can be misleading in the presence of class imbalance, additional metrics were examined. (ii) Precision quantified the model’s ability to avoid false-positive predictions, ensuring that normal samples were not incorrectly classified as tumors. (iii) Recall measured the proportion of true tumor cases that were correctly identified, and was critical for minimizing missed CRC diagnoses. (iv) The F1-score provided a balanced summary of precision and recall, which is particularly useful when both false positives and false negatives carry clinical consequences. (v) Area under curve (AUC-ROC), a threshold-independent metric of model discrimination, represents the probability that a randomly selected tumor sample would be ranked higher than a randomly selected normal sample. Receiver operating characteristic (ROC) curves were plotted to visualize the trade-off between sensitivity and specificity across the thresholds [37]. The graphical representation of these ROC curves is provided in Figure 2, Figure 3 and Figure 4, where curves approaching the upper-left corner and exhibiting higher AUC values indicate superior model performance. Details about the definitions and computation of the five performance metrics used to evaluate and compare the performances of the seven ML algorithms are provided in Appendix A.2.

#### 2.3.4. Model Selection

Having evaluated and compared the performances of the seven ML algorithms, we sought to select the most accurate ML algorithms for the discovery of potential clinically actionable biomarkers, therapeutic targets, and predictors of survival outcomes. All the trained models were evaluated using stratified 10-fold cross-validation (10-FCV). This approach preserved the tumor–normal distribution within each fold to reduce sampling bias. Performance assessment was based on accuracy, precision, recall, F1-score, and ROC–AUC, with all metrics averaged across folds to obtain stable estimates and mitigate variability. Additionally, because the dataset exhibited mild class imbalance, model ranking prioritized the F1-score and ROC–AUC, as these metrics provide a more informative balance between sensitivity and specificity in clinical classification tasks.

Following cross-validation, the model demonstrating the most favorable combination of discrimination ability, stability across folds, and computational efficiency was selected for downstream analyses. External validation using independent GEO datasets was incorporated into the selection process to ensure that the chosen classifier exhibited adequate generalization across heterogeneous platforms and experimental conditions. The final model chosen for interpretation was therefore the classifier that achieved a consistently strong performance across both internal and external evaluations while maintaining reproducibility and methodological robustness. The best-performing model was selected and subsequently used for feature/gene importance selection to address the second objective of this study, that is, leveraging the developed ML algorithms to discover potentially clinically actionable biomarkers, therapeutic targets, and predictors of survival outcomes.

#### 2.3.5. Feature or Gene Selection

The goal of feature (gene) selection was to prioritize candidate biomarkers and therapeutic targets, as well as to identify features with diagnostic and prognostic relevance. For each model, features/genes were identified using ML scores. Following the selection of the most accurate algorithms that were suitable for feature selection, a dedicated feature selection step was performed based on ML scores to identify the most informative genes for CRC classification. This was undertaken to ensure that the biomarker and target discovery were grounded in genes that were consistently identified across multiple ML algorithms. Using these approaches, the most accurate ML algorithm suitable for feature/gene selection was identified.

For feature/gene selection, we used three complementary approaches. The tree-based approach, a method used to capture nonlinear effects and higher-order interactions, the SHAP-based approach, a method which provided model-agnostic attribution of feature/gene contributions, and the coefficient-based approach, a linear model offering interpretable coefficient-based importance estimates. Details about the definition and computation of each feature selection strategy are provided in Appendix A.3. Each feature selection strategy was applied independently. This approach was undertaken to improve robustness, reproducibility, and biological interpretability by prioritizing genes that were consistently supported across multiple gene selection strategies [38].

Feature importance scores were computed within a 10-fold stratified cross-validation framework to ensure stability and minimize sampling bias. Fold-specific importance measures were aggregated to generate robust global rankings. Finally, gene ranks from all three feature selection strategies were combined to derive a consensus score, prioritizing genes that demonstrated consistent importance across independent methods. The resulting genes identified from each of the three feature section approaches were integrated into consensus gene sets using a three-way integration Venn diagram. Genes intersecting the three-way Venn diagram represented the consensus gene signature. These consensus genes were carried forward for downstream analysis, validation, functional enrichment analysis, network-based functional analysis, and prediction of survival outcomes, as described below. Detailed mathematical formulations and optimization procedures for all three feature selection methods are provided in Appendix A.3.

### 2.4. Integration of ML-Identified Genes with Somatic Mutation Information

Having identified genes using ML, we sought to investigate whether the ML-identified genes are somatic-mutated. The scientific premise was to infer the potential causal association between gene expression and the disease by determining whether ML-identified genes containing somatic mutations could be driving CRC tumorigenesis. To achieve this goal, we evaluated the ML-identified genes for the presence and number of somatic mutations per gene, and whether those somatic mutations mapped to the gene were pathogenic, as described earlier in this report. Note that somatic mutation data were not used as model input during training or feature selection; instead, they were analyzed post hoc. A gene was considered a potential driver if the mutation mapped to that gene and changed an amino acid. This integrative analysis connected expression-based classification with somatic alterations, providing a comprehensive view of the potential molecular drivers of CRC and reinforcing the biological credibility of the ML findings. Somatic mutated genes predictive of CRC were used in downstream analysis, including modeling protein–protein interactions, functional analysis, and predicting survival outcomes.

### 2.5. Modeling Protein–Protein Interaction Networks and Functional Enrichment Analysis

Having identified potential clinically actionable biomarkers and therapeutic targets using ML, and evaluated their ability to drive CRC tumorigenesis, we subjected the genes to protein–protein interaction (PPI) network modeling using a graph–theoretical centrality approach to map possible oncogenic interactions among the genes. Protein–protein interaction networks were constructed using STRING (v11.5), which integrates experimental evidence from curated databases, computational predictions, and text-mining sources [39]. High-confidence interactions supported by experimental data or strong predictive scores were retained. Details about the computational modeling of PPI are presented in Appendix A.4. To gain insights and characterize the molecular functions, biological processes, and cellular components in which ML-identified somatic mutated genes are involved, Gene Ontology (GO) analysis was performed. Additionally, we performed a KEGG pathways analysis to identify the signaling pathways they regulate.

### 2.6. Survival Analysis Using ML-Identified Genes

We performed an overall survival (OS) analysis to assess whether candidate diagnostic biomarkers and therapeutic targets enriched for somatic mutations identified by ML could be predictors of survival outcome. OS was defined as the time from initial diagnosis to death or last clinical follow-up for censored cases. Survival analyses were conducted on a gene-basis using Kaplan–Meier (KM) survival estimation [40], the log-rank test [41], and univariate Cox proportional hazards regression [42]. For each biomarker, patients were stratified into high- and low-expression groups using the median gene expression value as the cutoff. This median-based dichotomization resulted in near-balanced group sizes and provided a consistent, data-driven threshold for all genes.

Differences in survival distributions between high- and low-expression groups were evaluated using the log-rank test [34]. We used univariate Cox proportional hazards regression to quantify the magnitude and direction of associations between gene expression and OS [35]. The Cox model estimates the hazard ratio (HR), which represents the relative risk of death associated with higher gene expression. Survival differences between expression strata were evaluated using the log-rank test, reported as χ^2^ statistics and corresponding *p*-values, while Cox regression was used to estimate hazard ratios (HRs) with 95% confidence intervals (CIs) [41]. Using this approach, HR values less than 1 indicate a protective association with reduced mortality risk in the high-expression group, whereas HR values greater than 1 indicate an increased risk. HR values below 1 indicate a protective association with reduced mortality risk in the high-expression group, whereas HR values above 1 indicate increased risk [35]. We used the 95% intervals to reflect the precision of the HR estimates, and statistical significance was assessed using two-sided Wald tests [42].

## 3. Results

This study was conducted to develop and validate multi-model ML algorithms for the classification of tumor–normal samples using large-scale gene expression data, and to leverage the results from the developed ML algorithms to optimally integrate gene expression with somatic mutation information for the discovery of potential clinically actionable diagnostic biomarkers, therapeutic targets, and predictors of survival outcome. This section and subsequent subsections below present the results of the study.

### 3.1. Classification of Tumor Samples Using Multi-Model ML Algorithms

One of the primary objectives of this study was to develop and validate multi-model ML algorithms that could accurately classify tumor samples. The underlying hypothesis was that tumor–normal sample classification using ML would lead to early detection of CRC and identify individuals at high risk of developing aggressive disease, who could be prioritized for treatment. We addressed this objective by training, internally validating, and evaluating seven supervised ML models. The seven ML models included: RF, ET, GB, XGB, LGBM, SVM, and (LR). Model performances were assessed via stratified 10-fold cross-validation, and were evaluated using five performance metrics, namely: accuracy, precision, recall, F1 score, and area under the receiver operating characteristic curve (ROC–AUC). The results showing the performance of each of the seven models investigated using the test dataset and internal validation are presented in Table 2.

All seven ML classifiers demonstrated exceptionally strong internal performance, showing clear separation between tumor and normal CRC samples (Table 2). Among the seven models evaluated, RF, LR, XGB, and LGBM achieved perfect discrimination and the best performance, achieving 100% in accuracy, precision, recall, F1 score, and ROC–AUC (Table 2). The ET, GB, and SVM models also performed near-perfectly, achieving approximately 99.7% accuracy and F1 score, with recall and ROC–AUC values approaching 100.00% (Table 2). These results confirm our hypothesis that ML models could accurately classify tumor samples by effectively capturing gene expression signatures distinguishing CRC tumors from normal tissue.

While the high internal performance of ML models reflects strong model fitting, perfect scores can raise concerns regarding potential overfitting and the rigor and reproducibility of the models when evaluation is limited to a single dataset. In addition, internal validation alone is insufficient to establish the robustness, generalizability, and reproducibility of the models. To address this critical concern, all seven trained models were further evaluated using independent external datasets from GEO, as described in the Section 2, to ensure the rigor and reproducibility, robustness and generalizability of the models across heterogeneous data.

The results of this investigation are presented in Table 3. As evidenced from the results in the table, all seven models retained strong discriminative ability during external validation (Table 3). However, as expected, there were some reductions in the performance of the models on an independent dataset relative to their performance on the internal data. Among the seven models validated on independent datasets, RF, ET, and XGB emerged as the strongest performers, with each achieving 81.63% accuracy and perfect recall (100%), indicating excellent sensitivity to CRC tumor classification across heterogeneous datasets. Among the three models, RF achieved the highest ROC–AUC (88.44%) and the most balanced trade-off between precision, recall, and F1 score (86.57%), highlighting its robustness and reliability across different datasets. ET and XGB demonstrated nearly identical performance profiles, supporting the reproducibility of ensemble tree-based methods.

The SVM classifier also performed strongly, achieving 80.61% accuracy, 98.28% recall, and the highest ROC–AUC overall (89.35%), underscoring its strong discriminative capacity in high-dimensional data. However, its slightly lower precision suggests a modest increase in false-positive predictions relative to ensemble models. LR and LGBM showed competitive generalizability in performance, with accuracies exceeding 80% and recall values near 98%. This suggests that both linear and boosting-based models can generalize effectively when supported by robust preprocessing and threshold optimization. In contrast, GB exhibited the weakest external performance, with reduced accuracy, precision, and ROC–AUC, despite maintaining perfect recall (Table 3). The poor performance of the GB could partially be explained by the overfitting risk, which can occur if the model is not tuned properly (e.g., too many trees), in which case the model can overfit the training data. In this study the validation dataset was highly heterogeneous, which may have resulted in too many trees causing overfitting. Under such conditions the observed outcome is expected. This observation also highlights the need for validating ML algorithms on an independent dataset.

Taken together, across internal validation and independent external datasets, ML algorithms demonstrated strong and reproducible discrimination between tumor and normal samples. Validation using external heterogeneous GEO datasets, generated using different platforms and experimental protocols, supports the conclusion that the identified gene signature reflects stable, biology-driven expression patterns rather than dataset-specific technical artifacts. Such cross-platform generalizability is essential for clinical translation, particularly in the context of diagnostic assay development. Among all classifiers, RF demonstrated the most consistent and reliable performance across datasets, making it particularly well-suited for downstream analysis for feature selection. This investigation revealed that ML can accurately classify tumor samples and thus provides a framework for identifying individuals at high risk of developing aggressive disease, who could be prioritized for treatment to improve clinical outcomes. The lower performance of ML algorithms in the independent validation dataset compared to the perfect or near-perfect performance in the training dataset highlights the need for validating developed ML algorithms using independent datasets before general use.

### 3.2. Discovery of Potential Clinically Actionable Diagnostic Biomarkers and Therapeutic Targets

In addition to developing accurate classification models to distinguish tumors from normal tissue, a central objective of this study was to determine whether these algorithms could be leveraged for the discovery of potential clinically actionable features or diagnostic biomarkers. To address this goal, we implemented a multi-model feature selection framework integrating seven classifiers—RF, ET, XGBoost, LightGBM, GB, LR, and SVM. Because of the complexity and high dimensionality of transcriptomic data, no single model can fully capture all aspects of gene relevance. We therefore applied three complementary strategies for gene-importance evaluations: (i) tree-based feature importance derived from ensemble models, (ii) SHapley Additive exPlanations (SHAP) for model-agnostic interpretation, and (iii) coefficient-based importance from a linear SVM and Logistic Regression.

Each approach independently quantified the contribution of individual genes to model prediction; genes that were repeatedly ranked as important across these distinct analytical frameworks were prioritized as high-confidence candidate diagnostic biomarkers. This consensus-driven strategy was designed to maximize the robustness, reproducibility, and statistical reliability of the biomarker selection process. The results from each feature selection approach are summarized below.

#### 3.2.1. Tree-Based Feature Importance Identification Using Ensemble Models

The first feature selection strategy leveraged the intrinsic ranking capabilities of tree-based ensemble models, including bagging (RF, ET) and boosting (XGBoost, LightGBM, Gradient Boosting) approaches. Feature importance was quantified using impurity-based metrics for bagging models and gain-based metrics for boosting models, capturing both stable feature contributions and error-reduction efficiency. To enhance robustness, stratified 10-fold cross-validation was applied, and importance scores were averaged across folds. The resulting ranked gene list is provided in Table S1. The representative top-ranked genes identified by impurity-based and gain-based methods are summarized in Table 4a and Table 4b, respectively.

Following feature ranking, the importance scores derived from the five tree-based models were normalized and aggregated to generate a unified importance measure for each gene. Based on this integrated consensus ranking, the top 30 genes were selected, ensuring that feature prioritization reflects robust signals shared across multiple ensemble learning strategies rather than dependence on a single model.

To evaluate the independent diagnostic performance of these top 30 genes, each gene was assessed using a univariate Random Forest classifier with stratified 10-fold cross-validation (Figure 2). Random Forest was selected for this step due to its superior classification performance in the tumor–normal discrimination task. Importantly, it was used solely as a consistent evaluation model for ROC/AUC estimation, rather than as the basis for feature selection.

Most of the top 30 genes demonstrated excellent discriminative ability, with several—including *CADM3*, *GLP2R*, *CLEC3B*, *CDH3*, *TMEM151B*, *PLP1*, *MAMDC2*, *ETV4*, *TCF21*, *PINK1*, *PLCB4*, *ASPA*, *TMOD1*, *RNF112*, and *AC004707.1*—achieving near-perfect performance (AUC ≈ 0.99). Additional genes also showed strong diagnostic power (AUC ≈ 0.94–0.99), including *FAM161B*, *STBD1*, *VAMP2*, *DAAM2*, *PDE2A*, *CBX7*, *LDLRAD2*, *HAUS7*, *MIR497HG*, *MOCS1*, *ULBP3*, *CEMIP*, *RRAS2P1*, *MET*, and *ENC1*.

Together, tree-based importance and univariate ROC analysis provide complementary insights, identifying genes that are both critical within multivariate models and effective as independent diagnostic markers for CRC.

#### 3.2.2. SHAP-Based Model-Agnostic Feature Interpretation

The second feature selection strategy utilized SHAP (SHapley Additive exPlanations) values to obtain model-agnostic estimates of gene contributions across five ensemble classifiers (Extra Trees, Random Forest, Gradient Boosting, LightGBM, and XGBoost). SHAP assigns additive importance scores based on each gene’s marginal contribution to model predictions, enabling both global and sample-level interpretability. To enhance robustness, stratified 10-fold cross-validation was applied, and mean absolute SHAP values were computed and averaged across folds. The distribution of SHAP values for the top-ranked genes is shown in Figure S1, and the full SHAP-ranked gene list is provided in Table S2. Representative top-ranked genes identified across bagging and boosting models are summarized in Table 5a and Table 5b, respectively.

This analysis identified a consistent set of high-impact genes, including *CADM3*, *CDH3*, *CLEC3B*, *GLP2R*, *ETV4*, *GRIN2D*, and *TMEM151B*, which were repeatedly ranked among the top contributors across both bagging and boosting models.

Following feature ranking, SHAP importance scores derived from the five models were normalized and aggregated to generate a unified consensus importance measure for each gene. Based on this integrated ranking, the top 30 genes were selected, ensuring that feature prioritization reflects robust and reproducible signals across multiple modeling frameworks rather than dependence on a single classifier.

To evaluate the independent diagnostic performance of these genes, each was assessed using a univariate Random Forest classifier with stratified 10-fold cross-validation (Figure 3). Random Forest was selected for this step due to its superior classification performance in the tumor–normal discrimination task and was used solely as a consistent evaluation model for ROC/AUC estimation, rather than for feature selection.

Several genes demonstrated excellent discriminative ability (AUC ≥ 0.99), including *CADM3*, *GLP2R*, *CLEC3B*, *CDH3*, *TMEM151B*, *PLP1*, *MAMDC2*, *ETV4*, *TCF21*, *PINK1*, *PLCD4*, *ASPA*, *TMOD1*, *RNF112*, and *AC004707.1*, while others maintained strong performance (AUC ≈ 0.97–0.99). Even the lowest-performing gene (*ENC1*, AUC ≈ 0.94) retained substantial predictive power.

This two-stage framework—multi-model SHAP-based feature ranking followed by unified model evaluation—ensures robust biomarker identification, reduces model-specific bias, and enhances reproducibility.

#### 3.2.3. Coefficient-Based Feature Importance Using Linear Models

The third feature selection strategy utilized linear Support Vector Machines (SVM) and Logistic Regression (both with L2 regularization) to estimate gene importance based on model coefficients. Absolute coefficient values were extracted across stratified 10-fold cross-validation, averaged within each model, and used to generate a stable ranking of gene importance (Table S3). Representative top-ranked genes identified by the two linear models are summarized in Table 6.

This analysis identified several key genes, including *SIM2*, *COL11A1*, *KRT80*, *COL10A1*, *INHBA*, *WNT2*, *CST1*, *MMP7*, *EPHX4*, *GJB4*, *ESM1*, and *SST*, many of which overlapped with tree-based and SHAP-derived features, supporting their consistency across diverse modeling frameworks.

Following feature ranking, coefficient-based importance scores from both linear models were normalized and aggregated to generate a unified consensus importance measure for each gene. Based on this integrated ranking, the top 30 genes were selected, ensuring that feature prioritization reflects robust and reproducible signals rather than dependence on a single model.

To evaluate the independent diagnostic performance of these genes, each was assessed using a univariate Random Forest classifier with stratified 10-fold cross-validation (Figure 4). Random Forest was selected for this step due to its superior classification performance in the tumor–normal discrimination task and was used solely as a consistent evaluation model for ROC/AUC estimation, rather than for feature selection.

Several genes demonstrated strong discriminative ability, with *CDH3* and *KRT80* achieving near-perfect performance (AUC ≈ 0.99), followed by *CST1*, *MMP7*, and *GRIN2D* (AUC ≈ 0.96–0.97). Additional genes showed good performance (AUC ≈ 0.90–0.94), while a smaller subset exhibited moderate predictive ability (AUC ≈ 0.54–0.65), suggesting that these features may contribute more effectively within multigene signatures rather than as standalone markers.

This two-stage framework—coefficient-based feature ranking followed by unified Random Forest evaluation—ensures robust biomarker identification while reducing model-specific bias and enhancing reproducibility.

#### 3.2.4. Concordance Across Feature Selection Methods

To ensure a statistically rigorous comparison of feature importance across the three analytical strategies, we performed data-driven threshold optimization using an RF-based Top-N evaluation framework. Rather than selecting an arbitrary number of genes, we systematically evaluated thresholds of N = 50, 100, 150, 200, 250, and 300 using the meta-ranked feature sets derived from tree-based, SHAP-based, and coefficient-based approaches. For each threshold, reduced expression matrices were constructed and evaluated using strict 10-fold cross-validation, computing accuracy, F1-score, precision, recall, and ROC-AUC.

Predictive performance remained consistently high across thresholds from N = 50 to N = 250, with mean accuracy, precision, recall, and F1-score all ranging between 0.99 and 1.00, indicating near-perfect classification performance under cross-validation. A noticeable decline in performance was observed at N = 300, suggesting that the inclusion of lower-ranked genes introduces noise and reduces discriminative power. These trends are illustrated in Figure S2a–d. Based on this analysis, N = 250 was selected as the optimal threshold, representing a balance between model performance and feature comprehensiveness. While comparable performance was observed at lower thresholds (e.g., N = 150), the larger feature set at N = 250 enables the retention of additional informative genes without compromising predictive accuracy.

**Figure 5:**
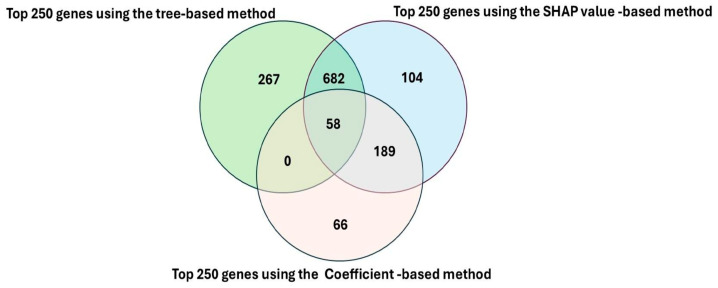
Three-way Venn diagram showing the overlap between genes identified by tree-based models, SHAP-based interpretation, and coefficient-based linear SVM/Logistic Regression. A consensus set of 58 genes was discovered by all three methods, reflecting their robustness and cross-model consistency.

The overlap between feature selection methods is illustrated in the Venn diagram (Figure 5). The largest pairwise overlap occurred between tree-based and SHAP-based approaches (682 genes), reflecting their shared ability to capture nonlinear and interaction-driven patterns. SHAP- and coefficient-based methods shared 189 genes, indicating that a subset of features contributes sufficiently to be detected by both nonlinear and linear models. Notably, the overlap between tree-based and coefficient-based methods was entirely encompassed within the three-way intersection.

The three-way intersection yielded a consensus signature of 58 genes, representing the most stable and reproducible biomarkers across all analytical strategies. These genes are robust to differences in model architecture and consistently identified across both linear and nonlinear frameworks, making them strong candidates for downstream biological interpretation and validation. In addition, each method contributed unique features (267 tree-based, 104 SHAP-based, and 66 coefficient-based), underscoring the complementary strengths of different modeling approaches in capturing distinct aspects of the transcriptomic landscape.

To investigate whether any of the ML-identified genes in the consensus 58 gene signature are implicated in CRC tumorigenesis, we performed in silico validation using published reports. The results showing the 58 gene signature, along with literature evidence support their implication in CRC, are presented in Table 7. In silico validation of the 58 gene signature using published reports revealed 51 genes reported to be involved in CRC and 7 novel genes (Table 7). These findings further confirmed that ML could accurately identify potential diagnostic biomarkers.

### 3.3. Discovery of a Signature of Potential Driver Genes

The development and progression of CRC involve the accumulation of somatic mutations. Thus, to address the hypothesis that ML-identified genes are somatic-mutated and likely potential drivers of CRC tumorigenesis, and could therefore serve as potential therapeutic targets, we evaluated the 58 genes identified by ML algorithms (Table 7) for the presence of somatic mutation frequency, as shown in Figure S3.

The results showing the number and distribution of somatic mutations among the 58 ML-identified genes are presented in Table 7. Out of the 58 genes identified by ML, 56 genes were somatic mutations, whereas 2 genes were not (Table 7). The most highly somatic-mutated genes were *COL11A1* (63 events), followed by *SPTBN2* (43), *GCNT2* (30), *ADAM12* (29), *SALL4* (27), and *CRIM1* (25) (Table 7). Additional genes, including GRIN2D, PHLPP2, SGK1, USP2, OPTN, and MRPL11, showed moderate mutation frequencies across patients (Table 4). In silico validation using published reports revealed that, among the somatic-mutated genes identified, all genes are implicated in CRC, whereas 7 genes were novel. Evaluation of somatic mutation for pathogenicity revealed that the most-detected genetic alterations were nonsynonymous single-nucleotide variants (missense, frameshift, or splice-site variants), which are known to affect protein structure and regulatory functions. The discovery of somatic mutations supports the interpretation that these genes are not only transcriptionally associated with CRC but could also be causally associated with the disease and may be contributing directly to CRC tumorigenesis.

The number and distribution of somatic mutations varied significantly. This can be partially explained by the clonal evolutionary dynamics involved in CRC development and progression. The absence of mutations in the genes *SMKR1* and *BLACAT1* does not diminish their biological or diagnostic relevance within a machine-learning-based framework, given that the development of many genes interacting with one another is demonstrated in the report on modeling protein–protein interactions. Overall, the investigation suggests that integrating somatic mutation information with ML-identified genes could identify potential drivers of CRC tumorigenesis, such as *COL11A1*, *SPTBN2*, *GCNT2*, *ADAM12*, and *SALL4.* If validated, such genes show promise as potential clinically actionable diagnostic biomarkers and targets for the development of novel, more effective therapeutics for CRC.

**Figure 6:**
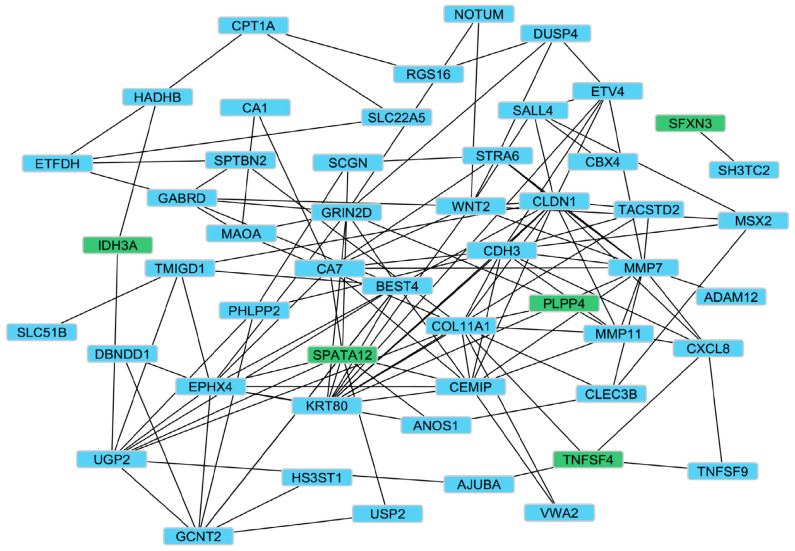
High-confidence protein–protein interaction (PPI) network constructed from the 58 potential CRC diagnostic biomarkers. Only genes with available somatic mutation counts were included in the visualization. Nodes represent biomarker genes and edges denote experimentally validated or strongly predicted interactions retrieved from the STRING database. Blue color nodes indicate somatic mutated genes that have been mentioned in the literature and green color indicates novel somatic-mutated genes.

To gain insights into the broader biological context of the 58 genes involved, we performed functional analysis modeling protein–protein interactions to determine whether the ML-identified somatic mutated genes interact. Our working hypothesis was that ML-identified somatic mutated genes interact and affect molecular networks, which s CRC tumorigenesis. To address this hypothesis, the 58 consensus diagnostic biomarkers enriched for somatic mutations identified by ML were mapped onto protein–protein interaction (PPI) architectures using the STRING database. The results showing the PPI networks for the ML-identified somatic mutated genes are presented in Figure 6.

PPI network analysis revealed dense functional connectivity with several central hubs, including *CDH3*, *COL11A1*, *WNT2*, *CLEC3B*, *CA7*, *SPATA12*, and *KRT80*, highlighting mutation-integrated interaction patterns that may contribute to CRC progression and therapeutic vulnerability. Network centrality metrics, including degree, betweenness, closeness, eigenvector centrality, and PageRank, further supported the identification of these key hub genes (Table S4). A number of genes with high mutation burdens, including *COL11A1*, *SPTBN2*, *GCNT2*, *ADAM12*, and *SALL4*, were centrally positioned or served as bridges between network submodules, suggesting that mutational events preferentially arise within structurally influential regions of the CRC interactome. The most highly somatic-mutated gene was *COL11A1*, which is localized within an ECM–adhesion–inflammation axis, whereas *SPTBN2* and *GCNT2* linked metabolic and signaling clusters, implying potential cross-pathway regulatory roles.

As evident in Figure 6, the systems-level network analysis further contextualized the diagnostic signature and potential therapeutic targeting within the CRC interactome. Most genes assembled into a densely connected network enriched for ECM organization, Wnt/β-catenin signaling, cytokine and chemokine communication, cell adhesion, and metabolic regulation, the hallmark processes of CRC tumorigenesis. Centrality analyses identified structurally dominant hubs such as *CLDN1*, *COL11A1*, *CDH3*, *KRT80*, *CEMIP*, *BEST4*, *WNT2*, *CA7*, and *SPATA12* [47,52,53,56,91]. Genes occupying such central positions are often key regulators of phenotypic state transitions, providing a mechanistic explanation for their potential as therapeutic targets. Interestingly, genes with fewer somatic mutations were found to interact with genes with highly mutated genes, confirming our hypothesis that CRC originates from a more complex interplay between constellations of genetic alterations (both rare and common variations) and potentially a broad range of environmental factors. These complex arrays of interacting genetic factors affect entire molecular networks that, in turn, increase or decrease the risk of disease or affect disease severity. The results of our network analysis in Figure 6 suggest that, in the context of CRC, the disease states can be considered emergent properties of molecular networks, as opposed to core biological processes associated with the disease, driven by responses to changes in a small number of genes. The results demonstrate that leveraging ML and integrating large-scale, high-dimensional molecular and somatic mutation data holds promise not only for defining the molecular networks regulating gene expression but also for causally associating such networks with the somatic mutations driving the disease.

To gain insights into the molecular functions, biological processes and cellular components in which ML-identified somatic mutated genes are involved, we performed a Gene Ontology (GO) analysis using the 58 genes. The top enriched GO terms included genes involved in glycosaminoglycan binding, heparin binding, carbon–oxygen lyase activity, hydro-lyase activity, and carbonate dehydratase activity (Figure S4). The most strongly enriched GO term, glycosaminoglycan binding, was of particular interest because glycosaminoglycans (GAGs) are major components of the extracellular matrix (ECM) and play critical roles in tumor invasion, metastasis, and modulation of the tumor microenvironment [82]. Dysregulated GAG protein interactions have been shown to promote CRC cell migration and enhance epithelial–mesenchymal transition (EMT) through pathways involving hyaluronan, proteoglycans, and ECM remodeling enzymes [92].

Heparin-binding proteins, including growth factors (e.g., VEGF, FGF), chemokines, and matrix-associated proteins, are known to regulate angiogenesis, inflammation, and proliferation in CRC, often driving aggressive tumor phenotypes and resistance to therapy [93]. The terms carbon–oxygen lyase activity and hydro-lyase activity reflect enzyme classes involved in metabolic and redox regulation. CRC tumors undergo profound metabolic reprogramming, relying on alterations in carbohydrate, amino acid, and lipid metabolism to sustain growth and adapt to hypoxic environments. Lyase activity has been implicated in the metabolic flexibility and modulation of the tumor acidic niche, both of which influence CRC progression and invasiveness [94].

Finally, enrichment of carbonate dehydratase activity, largely driven by the carbonic anhydrase family (such as *CA7*), aligns with the well-established role of carbonic anhydrases in regulating intracellular pH, extracellular acidification, and survival under hypoxia. Carbonic anhydrases promote CRC aggressiveness and metastasis and are being explored as promising diagnostic biomarkers and therapeutic targets [95].

Collectively, these enrichment and network analyses demonstrate that ML-identified diagnostic biomarkers and potential therapeutic targets converge on interconnected biochemical and signaling modules central to CRC pathophysiology. The integration of system-level topology, mutation burden, expression patterns, molecular function, and the interactions between novel genes and genes confirmed in the literature to be associated with CRC provides a mechanistic rationale for the diagnostic and therapeutic potential of both established and novel diagnostic biomarkers and therapeutic targets identified in this study.

### 3.4. Prediction of Survival Outcomes

**Figure 7:**
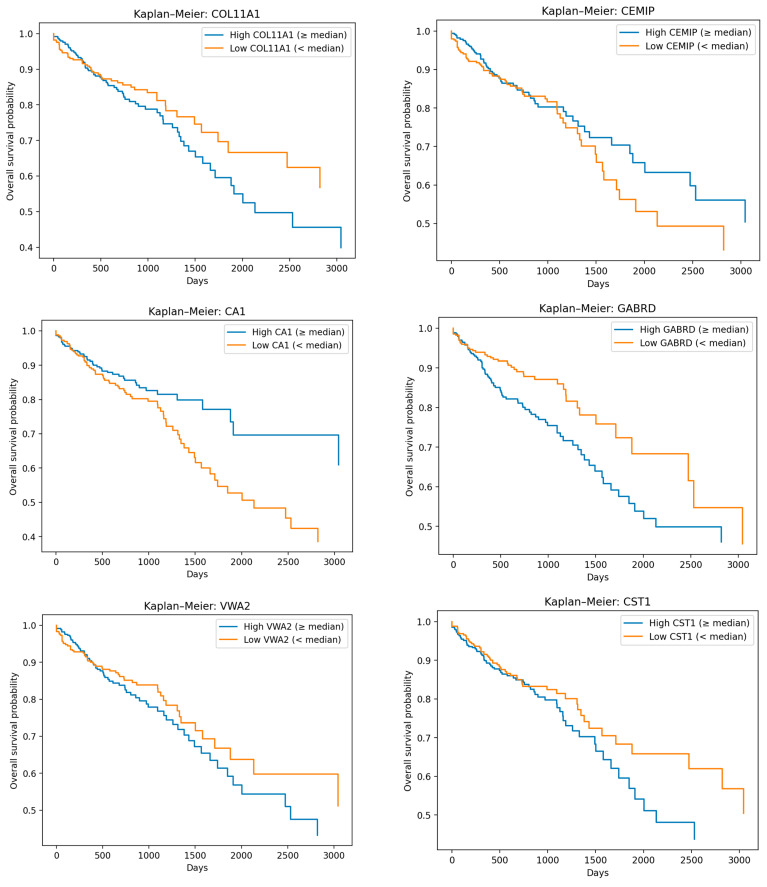

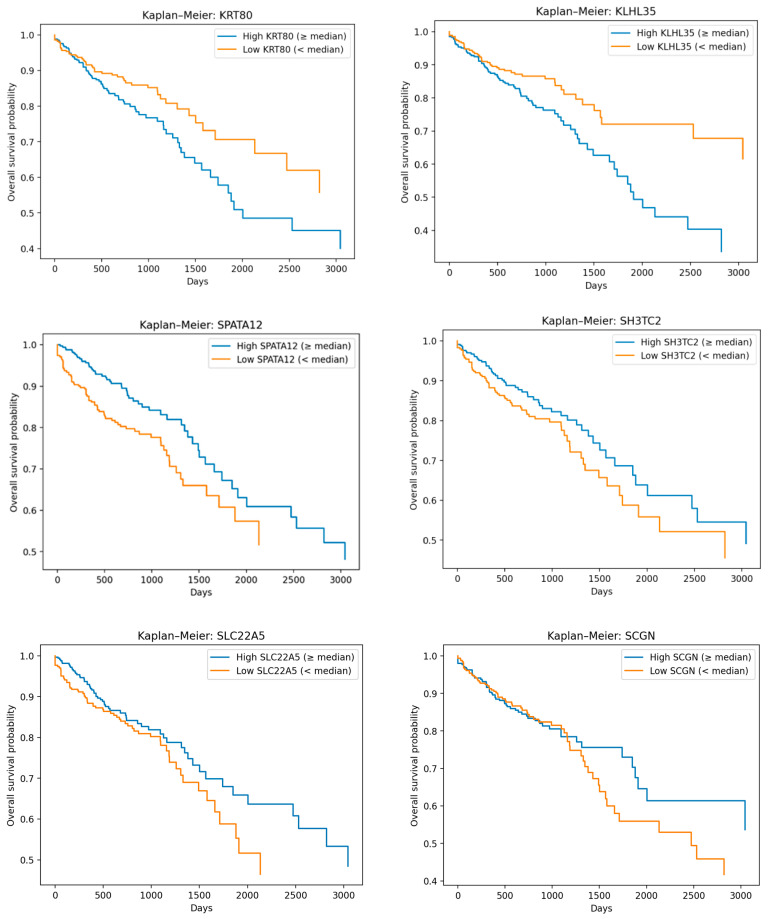
Kaplan–Meier plots showing 12 ML-identified genes that were predictors of overall survival. The figures include the somatic-mutated genes: *COL11A1*, *CEMIP*, *CA1*, *GABRD*, *VWA2*, *CST1*, *KRT80*, *KLHL35*, *SPARA12*, *SH3TC2*, *SLC22A5*, and *SCGN.* For each gene, patients were stratified into high- and low-expression groups using the median expression cutoff. The Kaplan–Meier curves depict OS probability over time (days), enabling visual assessment of survival differences between expression strata.

To determine whether the machine learning (ML)-identified somatically mutated genes possess prognostic relevance and can predict overall survival (OS), survival analyses were performed for all 58 genes identified through the ML framework, as described in the Section 2. Among the 58 genes analyzed, 33 were significantly associated with overall survival. The genes demonstrating the strongest prognostic value are presented in Figure 7, including *COL11A1*, *CEMIP*, *CA1*, *GABRD*, *VWA2*, *CST1*, *KRT80*, *KLHL35*, *SPATA12*, *SH3TC2*, *SLC22A5*, and *SCGN*.

Quantitative survival modeling revealed heterogeneous prognostic effects across the evaluated genes (Table S5). Among these, *SPATA12* demonstrated the strongest and most consistent association with patient outcome predictions. KM analysis showed a statistically significant difference between high- and low-expression groups (log-rank χ^2^ = 7.42, *p* = 0.0065). This finding was further supported by Cox regression, which indicated a significant reduction in mortality risk associated with higher *SPATA12* expression (HR = 0.772, 95% CI 0.613–0.973, *p* = 0.0283). Together, these results indicate that elevated *SPATA12* expression is associated with an approximately 23% decrease in hazard, supporting its role as a protective prognostic biomarker with potential clinical relevance.

In addition to the genes that were the best predictors of survival, other genes, including *ADAM12*, *CA7*, *CBX4*, *CDH3*, *CLDN1*, *CPT1A*, *DUSP4*, *HADHB*, *IDH3A*, *MMP11*, *PHLPP2*, *PLPP4*, *RMDN2*, *SPTBN2*, *STRA6*, *TACSTD2*, *TM1GD1*, *TNFSF4*, *USP2* and *SALL4*, predicted overall survival (Kaplan–Meier plots are shown in Document SD1). The ability of individual genes to predict overall survival varied. A number of genes did not accurately predict survival. However, as shown in the Figure showing protein–protein interactions, the genes that were predictive of survival outcome interacted with those that were not predictors of overall survival.

Overall, these findings suggest that ML-identified genes may serve as candidate biomarkers and therapeutic targets with potential diagnostic and prognostic value. Among them, *SPATA12* represents the most robust novel prognostic marker. The remaining genes show weaker or context-dependent effects in predicting outcome, highlighting the importance of integrating ML-derived biomarkers with survival modeling to identify candidates with both diagnostic and prognostic translational value. A number of genes were associated with an unfavorable survival outcome.

## 4. Discussion

We developed and validated a multi-model integrative analytical framework that incorporates multiple ML algorithms to accurately classify tumor–normal samples and leveraged the developed algorithms to discover clinically actionable diagnostic biomarkers, therapeutic targets, and predictors of survival outcome in CRC. ML algorithms accurately classified tumor–normal samples and identified a robust 58-gene signature.

The significance of these findings is that accurate tumor–normal classification provides a framework for identifying individuals who may be at high risk of developing aggressive disease and could be prioritized for treatment. Indeed, the use of ML for tumor–normal classification in CRC has been previously reported [18,56]. Several studies have demonstrated the effectiveness of ML algorithms, including Random Forest, Support Vector Machine, and gradient boosting methods, in accurately classifying tumor and normal samples and identifying diagnostic biomarkers [21,96,97]. These models are capable of capturing complex nonlinear gene expression patterns and have achieved high classification accuracy across multiple CRC datasets. Furthermore, integrative multi-omics studies combining transcriptomic and somatic mutation data have been shown to improve biomarker discovery and provide deeper biological insight into tumor progression and heterogeneity [20,21,57]. The novel aspect of our approach lies in the use of a multi-model integrative analytical framework that incorporates multiple ML algorithms for accurate tumor–normal classification and biomarker discovery. While prior studies have typically relied on single-model approaches or single data modalities, our framework integrates multiple complementary ML models and data types, thereby improving robustness, reducing model-specific bias, and enhancing the identification of clinically relevant biomarkers.

The discovery of the 58-gene signature using multi-model ML algorithms, which integrates gene expression with somatic information, was particularly significant. This finding demonstrates that the application of ML algorithms to large-scale, high-dimensional molecular and somatic mutation data holds promise not only for identifying individuals at high risk of aggressive disease but also for associating the ML-identified genes with genomic alterations driving CRC. Given the growing volume of data in CRC research, including single-cell RNA-seq, spatial transcriptomics, and epigenomics data, it is increasingly important to develop strategies for integrating these high-dimensional datasets using ML to better understand the molecular networks underlying disease states. Although we did not integrate epigenomics data in this study, our previous work has shown that DNA methylation can predict survival outcomes in CRC [98].

A major strength of this study lies in its integrative strategy for biomarker prioritization. Rather than relying on a single analytical perspective or purely differential expression analysis, we integrated somatic mutation with molecular profiling to construct causal probabilistic PPI networks of CRC. This approach provides a more comprehensive view of the molecular drivers of disease than analyzing each data type independently. Our PPI modeling and functional enrichment analysis demonstrate that CRC arises from a complex interplay among functionally related genes harboring constellations of genomic alterations. These interacting molecular components influence entire network states that, in turn, modulate disease risk and severity. Thus, CRC can be viewed as an emergent property of dysregulated molecular networks rather than the result of alterations in a limited number of individual genes.

Beyond a biological interpretation of molecular networks, these findings also provide a foundation for therapeutic innovation. In addition to biomarker discovery, the integration of machine learning-derived gene signatures with therapeutic modeling frameworks provides a compelling opportunity for AI-driven, multi-targeted drug discovery in colorectal cancer. Rather than focusing on single-gene targets, recent advances emphasize the identification of coordinated gene modules and network-level vulnerabilities that can be therapeutically exploited. Machine learning approaches can link disease-specific gene expression signatures with drug perturbation databases, such as Connectivity Map [99] and LINCS, to identify candidate compounds capable of reversing tumor-associated transcriptional states [59,60]. These strategies enable the systematic prioritization of drug repurposing candidates and rational combination therapies targeting multiple oncogenic pathways, including Wnt signaling, extracellular matrix remodeling, and metabolic reprogramming, which are central to CRC progression [92,100]. Furthermore, the predictive modeling of drug response using ML frameworks has demonstrated the ability to associate biomarker signatures with therapeutic sensitivity, thereby facilitating precision medicine approaches and improving treatment stratification [101]. Collectively, these advances highlight the potential of AI-guided, biomarker-driven strategies to accelerate the discovery of multi-targeted therapies and overcome drug resistance in CRC.

In parallel, increasing evidence suggests that microbial dysbiosis contributes to tumor initiation and progression through mechanisms involving inflammation, immune modulation, and metabolic reprogramming [102,103]. Multi-omics machine learning approaches integrating host transcriptomics with microbiome-derived metagenomic and metabolomic data have enabled the identification of composite biomarkers that capture both host and microbial contributions to disease [104]. Notably, specific microbial taxa, including *Fusobacterium nucleatum*, have been consistently associated with CRC and may influence tumor behavior and therapeutic response [105,106]. Integrating host–microbiome interactions into future ML frameworks may enhance early detection, risk stratification, and therapeutic targeting, particularly in early-onset colorectal cancer.

From a clinical perspective, effective colorectal cancer prevention relies heavily on the screening strategies implemented by healthcare personnel. Established approaches, including colonoscopy and fecal immunochemical testing (FIT), remain essential for early detection and reducing mortality [107,108]. Healthcare providers play a critical role in identifying at-risk individuals, promoting adherence to screening programs, and ensuring timely follow-up. The integration of machine learning-based risk prediction models into clinical workflows may further enhance screening efficiency by identifying individuals who could benefit from earlier or more frequent surveillance [109].

Recent advances in deep learning have significantly enhanced the diagnostic landscape of colorectal cancer, particularly in histopathological and endoscopic image analysis. Deep learning models, including convolutional neural networks (CNNs), have demonstrated high accuracy in detecting and classifying colorectal lesions from whole-slide histopathology images, improving diagnostic precision and reducing inter-observer variability [110,111]. In addition, deep learning approaches have been successfully applied to real-time polyp detection during colonoscopy [112]. The integration of imaging-based deep learning with molecular machine learning frameworks offers a promising multi-modal strategy for improving CRC diagnosis and clinical decision-making.

Complementing imaging-based advances, the identification of novel biomarkers remains critical for improving colorectal cancer diagnosis and therapeutic targeting. Among emerging candidates, butyrylcholinesterase (BChE) has been associated with metabolic and inflammatory processes linked to tumor progression [113]. Altered levels of BChE have been reported in cancer patients and may reflect tumor-associated metabolic dysregulation. Integrating such emerging biomarkers with gene expression-based ML frameworks may enhance diagnostic accuracy and support personalized therapeutic strategies.

### Translational Impact

The findings of this study have several translational implications for clinical practice in colorectal cancer management. Particular attention is given to how multi-model integrative ML modeling can link molecular states to genetic alterations, thereby identifying diagnostic biomarkers and therapeutic targets and complementing existing clinical protocols.

(i) ML Classification of Tumor–Normal Samples: Accurate classification using ML algorithms can aid in therapeutic decision-making by stratifying patients and identifying individuals at high risk of aggressive disease. Additionally, ML approaches may support screening and early detection, which are critical given the poor prognosis associated with late-stage CRC diagnosis.

(ii) ML-Identified Diagnostic Biomarkers and Therapeutic Targets: In this study, we identified a 58-gene signature using ML algorithms. All 58 genes in the signature harbor somatic mutations, the majority of which are missense mutations that alter amino acid sequences. Through in silico validation, many of the identified genes have been previously implicated in CRC (see Table 4 with references therein), while others represent novel findings.

Many of the 58 genes have well-established roles in CRC development and progression. Prominent examples include *CDH3*, *COL11A1*, *CEMIP*, *CLDN1*, *MMP7*, *MMP11*, *WNT2*, *KRT80*, and *CXCL8*, which are implicated in epithelial–mesenchymal transition, extracellular matrix (ECM) remodeling, invasion, angiogenesis, immune modulation, and adverse clinical outcomes [114,115,116,117,118]. In addition to established CRC-associated genes, the diagnostic signature highlights metabolic and mitochondrial regulators such as *CA1*, *CA7*, *CPT1A*, *ETFDH*, *HADHB*, *IDH3A*, and *SFXN3* [119,120,121,122]. Notably, this study also identified several genes with limited or no prior annotation in CRC, including *PLPP4*, *RMDN2*, *SH2D7*, *SMKR1*, *SPATA12*, and TNFSF4. Their recurrent prioritization across independent analytical strategies suggests that they represent genuine and previously underrecognized components of CRC biology. Several of these genes occupied influential positions within the PPI network, further supporting their potential functional relevance.

Through functional enrichment analysis, extracellular matrix organization, glycosaminoglycan-mediated interactions, and metabolic regulation pathways were strongly associated with these genes, including integrin signaling and MAPK signaling pathways. The cell cycle plays a central role in cancer development, and dysregulated genes may promote tumor progression by influencing cell cycle control [123].

Gene Ontology (GO) analysis revealed significant enrichment in molecular functions such as glycosaminoglycan binding, heparin binding, carbonate dehydratase activity, and lyase-related enzymatic functions. These findings highlight the importance of ECM remodeling, growth factor signaling, metabolic plasticity, and pH regulation in CRC. The emergence of these pathways from an unbiased, data-driven gene set supports the biological coherence of the identified signature.

Extracellular matrix organization is tightly linked to tumor progression and microenvironmental dynamics. The ECM provides structural support, regulates signaling pathways, and influences cellular behavior. During tumorigenesis, interactions between cancer cells and the tumor microenvironment often result in ECM remodeling and stiffening, which can promote malignant transformation and disease progression.

Taken together, these findings support the hypothesis that the identified 58-gene signature, if experimentally validated, could serve as a source of diagnostic biomarkers and therapeutic targets. Furthermore, the identification of genes associated with survival outcomes suggests their potential utility as prognostic biomarkers.

(iii) Complementarity of ML Findings to Existing Protocols: Our study accurately classified tumors and identified clinically relevant diagnostic and prognostic biomarkers, highlighting the translational potential of these findings. While colonoscopy remains the primary tool for colorectal cancer diagnosis [107], it has limitations. These include reduced effectiveness in certain populations, the need for follow-up procedures in some cases, and limited ability to identify individuals at high risk of aggressive disease. Importantly, colonoscopy does not provide insights into the molecular drivers of CRC.

The ML-identified biomarkers can complement colonoscopy by providing molecular-level information that supports more precise diagnosis. For patients already diagnosed with CRC, monitoring the expression of specific biomarkers may provide deeper insights into disease progression and enable more personalized treatment strategies.

Thus, integrating ML-derived biomarkers into clinical workflows may enhance diagnostic accuracy, improve patient stratification, and support precision oncology approaches. We anticipate that this study, along with future investigations, will contribute to the development of more effective, biomarker-driven clinical protocols for CRC.

Limitations of the Study: The study provides valuable insights into the application of ML to the classification of tumors, the discovery of potential clinically actionable biomarkers, therapeutic targets, and predictors of survival outcome in CRC. However, limitations of the study must be acknowledged. This study was limited to integrating gene expression with somatic mutation data. The development and progression of CRC involves a complex interplay between genes and the environment. Therefore, future studies should consider integrating genomics with epigenomic and single-cell RNA-Seq to understand how genomic and epigenomic alterations influence the cellular states to drive CRC. Second, although rigorous cross-platform validation was performed, a prospective validation in clinically annotated, multi-center cohorts using standardized sequencing pipelines is required prior to clinical deployment. Third, additional molecular layers, including copy-number variation, proteomics, metabolomics, and microbiome profiles, were not incorporated and may further refine biomarker selection and mechanistic insight. Finally, experimental validation is necessary to confirm the functional roles and therapeutic tractability of several novel candidates identified herein. These limitations were beyond the scope of this study but will be future areas of research. Notwithstanding these limitations, this study demonstrates the power of using multi-model integrative ML algorithms for the classification of tumor–normal samples and the discovery of potential clinically actionable biomarkers, therapeutic targets, and predictors of survival outcome in CRC.

## 5. Conclusions

The study demonstrates that multi-model integrative ML algorithms using gene expression data and somatic mutation information can accurately classify tumor–normal samples in CRC. The study further demonstrates that multi-model integrative ML can be leveraged to optimally integrate gene expression data with somatic mutation information for the discovery of potential clinically actionable biomarkers and therapeutic targets, and predictors of survival outcome in CRC. Overall, the study demonstrates that multi-model integrative ML provides a powerful approach for harnessing large-scale multi-omics data for tumor–normal classification and the discovery of potential diagnostic biomarkers, therapeutic targets, and predictors of outcomes to aid in the clinical management of CRC and improve outcomes.

## Figures and Tables

**Table 1:** Sources and distribution of datasets used for model training, testing, and external validation.

Dataset Type	Dataset ID	Tumor	Normal	Total
Training and Testing	TCGA	989	54	1043
	GTEx	-	822	822
	Total	989	876	1865
External Validation	Dataset ID	Tumor	Normal	Total
	GSE251845	22	22	44
	GSE50760	36	18	54
	Total	58	40	98

**Table 2:** Performance metrics of various machine learning models for colorectal cancer classification using RNA-Seq data.

Model Type	Accuracy	Precision	Recall	F1 Score	ROC-AUC	Threshold
Random Forest (RF)	100.00%	100.00%	100.00%	100.00%	100.00%	0.608
Extra Trees (ET)	99.73%	99.50%	100.00%	99.75%	100.00%	0.513
Gradient Boosting (GB)	99.73%	99.50%	100.00%	99.75%	100.00%	0.051
Logistic Regression (LR)	100.00%	100.00%	100.00%	100.00%	100.00%	0.258
XGBoost (XGB)	100.00%	100.00%	100.00%	100.00%	100.00%	0.645
LightGBM (LGBM)	100.00%	100.00%	100.00%	100.00%	100.00%	0.606
Support Vector Machine (SVM)	99.73%	99.50%	100.00%	99.75%	99.99%	0.522

**Table 3:** External validation performance of ML models on independent GEO colorectal cancer datasets. (The best performing models in bold fonts.)

Model Type	Accuracy	Precision	Recall	F1 Score	ROC-AUC	Threshold
**Random Forest (RF)**	**81.63%**	76.31%	**100%**	**86.57%**	**88.44%**	0.608
Extra Trees (ET)	81.63%	**76.32%**	100%	86.57%	85.26%	0.513
Gradient Boosting (GB)	75.51%	70.73%	100%	82.86%	79.83%	0.051
Logistic Regression (LR)	80.61%	76.00%	98.28%	85.71%	85.82%	0.258
XGBoost (XGB)	81.63%	**76.32%**	100%	86.57%	85.60%	0.645
LightGBM (LGBM)	80.61%	76.00%	98.28%	85.71%	85.60%	0.606
Support Vector Machine (SVM)	80.61%	76.00%	98.28%	85.71%	89.35%	0.522

**Table 4:** (**a**) Top-ranked genes identified by impurity-based tree ensemble models for colorectal cancer (CRC) classification. The table presents genes prioritized independently by Random Forest (RF) and Extra Trees (ET), along with their chromosomal locations (Chr_Pos) and feature importance scores (Imp). (**b**) Top-ranked genes identified by gain-based tree ensemble models for colorectal cancer (CRC) classification. The table summarizes genes selected independently by Gradient Boosting (GB), LightGBM (LGBM), and XGBoost (XGB), with their chromosomal locations (Chr_Pos) and corresponding feature importance scores (Imp).

RF_Gene	Chr_Pos	Imp	ET_Gene	Chr_Pos	Imp	XGB_Gene	Chr_Pos	Imp
(**a**)								
*FAM161B*	14q24.3	0.015379	*ARL6IP4*	12q24.31	0.00720			
*PINK1*	1p36.12	0.011131	*AC090004.1*	1q23.1	0.00593			
*LDLRAD2*	1p36.12	0.010892	*ST13P4*	22q13.31	0.00574			
*MIR497HG*	17p13.1	0.01056	*MED4.AS1*	13q14.2	0.00573			
*CLEC3B*	3p21.31	0.008673	*RPL23AP42*	3q26.1	0.00567			
(**b**)								
*CADM3*	1q23.2	0.97516	*CDH3*	16q22.1	79.5	*CADM3*	1q23.2	0.32417
*GRIN2D*	19q13.33	0.01620	*CADM3*	1q23.2	57.3	*CDH3*	16q22.1	0.15089
*ENC1*	5q13.3	0.00148	*GLP2R*	17p13.1	39.1	*CLEC3B*	3p21.31	0.15051
*ETV4*	17q21.31	0.00107	*PLPP1*	5q11.2	31.9	*GLP2R*	17p13.1	0.08168
*IDH3A*	15q25.1	0.00103	*ETV4*	17q21.31	26	*PLPP1*	5q11.2	0.04355

**Table 5:** (**a**) Top-ranked genes identified using SHAP-based feature importance for colorectal cancer (CRC) classification. The table presents genes prioritized independently by Random Forest (RF) and Extra Trees (ET) models, along with their chromosomal locations (Chr_Pos) and mean absolute SHAP importance scores (Imp). (**b**) Top-ranked genes identified using SHAP-based feature importance for colorectal cancer (CRC) classification. The table summarizes genes selected independently by Gradient Boosting (GB), LightGBM (LGBM), and XGBoost (XGB) models, together with their chromosomal locations (Chr_Pos) and mean absolute SHAP importance scores (Imp).

RF_Gene	Chr_Pos	Imp	ET_Gene	Chr_Pos	Imp	XGB_Gene	Chr_Pos	Imp
(**a**)								
*FAM161B*	14q24.3	0.00578	*ARL6IP4*	12q24.31	0.00322			
*MIR497HG*	17p13.1	0.00475	*RPL23AP42*	3q26.1	0.00266			
*PINK1*	1p36.12	0.00461	*AC090004.1*	1q23.1	0.00264			
*LDLRAD2*	1p36.12	0.00459	*MED4.AS1*	13q14.2	0.00254			
*CDH3*	16q22.1	0.00375	*AC023055.1*	7p21.3	0.00249			
(**b**)								
*CADM3*	1q23.2	8.2412	*CADM3*	1q23.2	1.4008	*CADM3*	1q23.2	2.32417
*GRIN2D*	19q13.33	1.6784	*GLP2R*	17p13.1	0.9547	*CDH3*	16q22.1	0.32089
*ETV4*	17q21.31	0.1279	*CDH3*	16q22.1	0.8882	*CLEC3B*	3p21.31	0.23401
*CDH3*	16q22.1	0.1089	*CLEC3B*	3p21.31	0.6460	*GLP2R*	17p13.1	0.06168
*ENC1*	5q13.3	0.0923	*PLPP1*	5q11.2	0.5603	*PLPP1*	5q11.2	0.04623

**Table 6:** Top-ranked genes identified using coefficient-based feature importance for colorectal cancer (CRC) classification. The table presents genes prioritized independently by Logistic Regression (LR) and linear Support Vector Machine (SVM) models, along with their chromosomal locations (Chr_Pos) and absolute coefficient values (Imp).

LR_Gene	Chr_Pos	Imp	SVM_Gene	Chr_Pos	Imp
*SIM2*	21q22.13	0.01030	*COL11A1*	1p21.1	0.00170
*COL11A1*	1p21.1	0.00955	*SIM2*	21q22.13	0.00169
*KRT80*	12q13.13	0.00891	*KRT80*	12q13.13	0.00154
*WNT2*	7q31.2	0.00879	*COL10A1*	6q22.1	0.00142
*INHBA*	7p14.1	0.00876	*INHBA*	7p14.1	0.00140

**Table 7:** List of the 58 consensus genes identified by the three complementary feature selection approaches (tree-, SHAP- and coefficient-based) approaches, along with the number of somatic mutations and references indicating association with CRC.

Gene Name	Chromosome Position	Total_Mutations	Reference Cited
*AC021218.1*	Long non-coding	1	[43]
*ADAM12*	10q26.2	29	[44]
*AJUBA*	14q11.2	4	[45]
*ANOS1*	Xp22.31	17	[46]
*BEST4*	1p34.1	10	[47]
*BLACAT1*	1q32.1	0	[48]
*CA1*	8q21.2	4	[49]
*CA7*	16q22.1	7	[50]
*CBX4*	17q25.3	11	[51]
*CDH3*	16q22.1	9	[52]
*CEMIP*	15q25.1	25	[53]
*CLDN1*	3q28	1	[54]
*CLEC3B*	3p21.31	6	[55]
*COL11A1*	1p21.1	63	[56]
*CPT1A*	11q13.3	17	[57]
*CST1*	20p11.21	10	[58]
*CXCL8*	4q13.3	4	[59]
*DBNDD1*	11q24.2	4	[60]
*DUSP4*	8p12	10	[61]
*EPHX4*	1p36.21	9	[62]
*ETFDH*	4q32.1	8	[63]
*ETV4*	17q21.31	11	[64]
*GABRD*	1p36.33	12	[65]
*GCNT2*	6p24.2	30	[66]
*GRIN2D*	19q13.33	18	[67]
*HADHB*	2p23.3	12	[67]
*HS3ST1*	4q25	8	[68]
*IDH3A*	19p13.12	6	Novel
*KLHL35*	16q22.1	5	[69]
*KRT80*	12q13.13	6	[70]
*MAOA*	Xp11.3	5	[71]
*MMP11*	22q11.23	15	[72]
*MMP7*	11q22.2	9	[73]
*MSX2*	5q35.2	8	[74]
*NOTUM*	17p13.1	9	[75]
*PHLPP2*	16q22.1	18	[76]
*PLPP4*	10q23.2	7	Novel
*RGS16*	1q25.3	3	[77]
*RMDN2*	19q13.33	12	Novel
*SALL4*	20q13.13	27	[78]
*SCGN*	6p22.2	7	[79]
*SFXN3*	10q24.31	5	Novel
*SH2D7*	Xp11.4	3	[80]
*SH3TC2*	5q32	18	[81]
*SLC22A5*	5q31.1	8	[82]
*SLC51B*	15q22.31	1	Novel
*SMKR1*	**7q32.1**	0	Novel
*SPATA12*	3p14.3	3	Novel
*SPTBN2*	11q13.2	43	[83]
*STRA6*	15q24.1	9	[84]
*TACSTD2*	1p32.1	5	[85]
*TMIGD1*	17q11.2	9	[86]
*TNFSF4*	1q25.1	10	Novel
*TNFSF9*	19p13.3	6	[87]
*UGP2*	2p15	14	[88]
*USP2*	11q23.3	17	[89]
*VWA2*	10q25.3	15	[90]
*WNT2*	7q31.2	13	[91]

“Novel” indicates that, no previously published studies have reported or investigated the association of this specific gene with colorectal cancer (CRC).

## Data Availability

Original RNA-Seq, somatic mutation, and clinical data used in this study are available and downloadable at the Genomics Data Commons (GDC) website: https://gdc.cancer.gov/ (accessed on 20 June 2025). Original data used for validation of ML models is available and downloadable at the Gene Expression Omnibus (GEO) website: https://www.ncbi.nlm.nih.gov/geo/ (accessed on 20 November 2025). Supplementary Data contained in this manuscript is available at https://www.mdpi.com/ethics (accessed on 9 March 2026).

## Supplementary Materials

The following supporting information can be downloaded at: https://www.mdpi.com/article/10.3390/cancers18101503/s1: Figure S1. SHAP summary plot illustrates the contribution of the top 30 genes to CRC classification. Figure S2. Performance of the Random Forest classifier as a function of the Top-N intersection gene threshold. Figure S3. Somatic mutation frequencies among the 58-machine learning-derived diagnostic biomarkers using TCGA CRC whole-exome sequencing data. Figure S4. Molecular Function (MF) enrichment analysis for the 58 diagnostic biomarker genes. Table S1. Ranked genes identified by tree-based ensemble models for colorectal cancer (CRC) classification, accompanied by their chromosomal locations and model-specific feature importance scores. Table S2. Genes ranked by SHAP-based feature importance aggregated across five ensemble machine learning models. Table S3. Genes ranked by mean absolute coefficients derived from L2-regularized Linear Support Vector Machine (SVM) and Logistic Regression (LR) models. Table S4. Network centrality metrics for the diagnostic biomarker gene panel identified in colorectal cancer (CRC). Table S5. Overall survival (OS) analysis of machine learning-identified novel biomarkers using Kaplan–Meier (KM) and univariate Cox regression. Document SD1. Kaplan–Meier plots showing additional ML-identified genes that were predictive of survival outcome.

## Abbreviations

The following abbreviations are used in this manuscript:

AI	Artificial Intelligence
AUC	Area Under the Curve
CI	Confidence Interval
CRC	Colorectal Cancer
DEL	Deletion
DNA	Deoxyribonucleic Acid
ECM	Extracellular Matrix
EMT	Epithelial–Mesenchymal Transition
ET	Extra Trees
FCV	Fold Cross-Validation
F1-score	Harmonic Mean of Precision and Recall
FOBT	Fecal Occult Blood Test
GB	Gradient Boosting
GDC	Genomics Data Commons
GEO	Gene Expression Omnibus
GO	Gene Ontology
GTEx	Genotype-Tissue Expression
HR	Hazard Ratio
INS	Insertion
KM	Kaplan–Meier
LGBM	Light Gradient Boosting Machine
LR	Logistic Regression
MAF	Mutation Annotation Format
ML	Machine Learning
OS	Overall Survival
PPI	Protein–Protein Interaction
RF	Random Forest
ROC	Receiver Operating Characteristic
RNA-Seq	RNA Sequencing
SHAP	SHapley Additive exPlanations
SNP	Single Nucleotide Polymorphism
SVM	Support Vector Machine
TCGA	The Cancer Genome Atlas
US	United States
XGB	Extreme Gradient Boosting
χ^2^	Chi-square Statistic

## Appendix A

This Supplementary Methods document includes additional details about the Methods and supportive or illustrative figures providing detailed mathematical formulations, extended methodological descriptions about ML models used (Appendix A.1), computational performance metrics (Appendix A.2), descriptions and computational methods related to feature/gene selection (Appendix A.3), and computational methods for protein–protein interaction modeling (Appendix A.4), along with the cited references.

### Appendix A.1. Formulation and Computation of Machine-Learning Models

This section describes the computational framework for the ML methods used in the study. Random Forest (RF) and Extra Trees (ET), both ensemble-based methods, were applied to enhance model stability and reduce overfitting through ensemble learning [124]. These models collectively represent a spectrum of learning strategies—ranging from linear decision boundaries to ensemble tree-based learners and gradient-boosted frameworks—allowing the study to characterize the heterogeneous gene-expression signatures associated with CRC and to identify robust diagnostic biomarkers. In RF, each decision tree $h_{b}(x)$ is trained on a bootstrap sample, and the final prediction is obtained by averaging across all B trees, as shown in Equation (A1):

(A1) $$\hat{y}=\frac{1}{B}\sum_{b=1}^{B}h_{b}(x)$$

where $\hat{y}$ is the predicted class probability, B is the number of trees, and x is the gene-expression vector. Tree splits were optimized using the Gini impurity criterion, defined in Equation (A2):

(A2) $$G(t)=\sum_{k=1}^{K}p_{k}(t)[1-p_{k}(t)],$$

where G(t) is the impurity of node t, K is the number of classes (tumor vs. normal), and $p_{k}(t)$ is the proportion of samples at node t belonging to class k. The reduction in impurity achieved by a split is computed as in Equation (A3):

(A3) $$\Delta G(t)=G(t)-\sum_{m\in \{L,R\}}\frac{N_{m}}{N_{t}}\;G(m),$$

where $N_{t}$ is the number of samples in the parent node and $N_{m}\;$represents the number of samples routed to the left (L) or right (R) child. Extra Trees (ET) increases randomization by selecting random thresholds θ for candidate feature splits of the form $t=\{x|x_{j}<\theta \}$, producing decorrelated trees that perform well in ultra-high-dimensional gene-expression settings.

Boosting-based models—GB [32], XGBoost [33], and LightGBM [34]—were integrated to construct strong learners by iteratively correcting errors made by previous weak learners. Gradient Boosting builds an additive model where the ensemble prediction after iteration m follows Equation (A4):

(A4) $$F_{m}(x)=F_{m-1}(x)+\nu \;h_{m}(x),$$

where $F_{m-1}(x)$ is the previous prediction, $h_{m}(x)$ is the new weak learner (a shallow regression tree), and ν is the learning-rate hyperparameter. Each new learner fits the negative gradient of the chosen loss function with respect to the current model predictions, as in Equation (A5):

(A5) $$g_{i}^{\left(m\right)}=-{\left[\frac{\partial L(y_{i},F(x_{i}))}{\partial F(x_{i})}\right]}_{F=F_{m-1}},$$

where $g_{i}^{\left(m\right)}$ is the pseudo-residual, L is the differentiable loss (binary cross-entropy), $y_{i}$ is the true label, and $F(x_{i})$ is the model score.

XGBoost extends gradient boosting by optimizing a regularized objective function in Equation (A6):

(A6) $$L=\sum_{i=1}^{n}l(y_{i},{\hat{y}}_{i})+\sum_{k=1}^{K}\Omega (f_{k}),$$

where n is the number of samples, $l(y_{i},{\hat{y}}_{i})$ is the pointwise loss, $f_{k}$ is the k-th tree, and K is the number of boosting rounds. The regularization term $\Omega (f_{k})$ is defined in Equation (A7):

(A7) $$\Omega (f_{k})=\gamma T+\frac{1}{2}\lambda \sum_{j=1}^{T}w_{j}^{2},$$

where T denotes the number of leaves, $w_{j}$ is the leaf weight, and γ and λ penalize tree complexity via leaf count and L2 weight regularization, respectively. LightGBM improves efficiency by growing trees leaf-wise rather than level-wise and by leveraging first- and second-order gradient statistics. The gain from a split is computed using Equation (A8):

(A8) $$\mathrm{Gain}=\frac{1}{2}(\frac{G_{L}^{2}}{H_{L}+\lambda }+\frac{G_{R}^{2}}{H_{R}+\lambda }-\frac{(G_{L}+G_{R}{)}^{2}}{H_{L}+H_{R}+\lambda })-\gamma ,$$

where $G_{L}$ and $G_{R}$ are sums of first-order gradients, $H_{L}$ and $H_{R}$ are sums of second-order gradients (Hessians), λ is an L2 regularization term, and γ penalizes split complexity.

Logistic Regression with L2 regularization served as a linear and interpretable benchmark model [35]. Class probabilities were computed using the logistic sigmoid function given in Equation (A9):

(A9) $$\hat{y}=\sigma (w^{⊤}x+b)=\frac{1}{1+e^{-(w^{⊤}x+b)}},$$

where x is the expression vector, w is the coefficient vector, and b is the intercept. To mitigate overfitting and stabilize coefficient estimates in high-dimensional settings, the model minimized the L2-regularized loss shown in Equation (A10):

(A10) $$L(w,b)=-\sum_{i=1}^{n}[y_{i}log({\hat{y}}_{i})+(1-y_{i})log(1-{\hat{y}}_{i})]+\lambda ∥w{∥}_{2}^{2},$$

where $∥w{∥}_{2}^{2}=\sum_{j=1}^{p}w_{j}^{2}\;$is the squared L2 norm and λ controls regularization strength. Each coefficient $w_{j}$ indicates the strength and direction of association between gene j and tumor phenotype, providing built-in interpretability.

Support Vector Machines (SVMs) were incorporated due to their effectiveness in high-dimensional spaces [31]. The linear SVM seeks a separating hyperplane described in Equation (A11):

(A11) $$w^{⊤}x+b=0,$$

where w defines the hyperplane orientation and b is the bias. The optimization objective maximizes the margin while allowing soft-margin violations, as formulated in Equation (A12):

(A12) $$\underset{w,b,\{\xi_{i}\}}{min}\frac{1}{2}∥w{∥}_{2}^{2}+C\sum_{i=1}^{n}\xi_{i},\;\mathrm{subject}\;\mathrm{to}\;y_{i}(w^{⊤}x_{i}+b)\geq 1-\xi_{i},\;\xi_{i}\geq 0,$$

where C balances margin width and misclassification penalties. All models were trained using consistent preprocessing, hyperparameter tuning, and evaluation procedures to ensure fair comparison. The integration of linear models (LR and SVM), nonlinear ensemble learners (RF and ET), and gradient-based boosting algorithms (XGBoost, LightGBM, and GB) provided a comprehensive and complementary analytical framework. This multi-model strategy captured diverse biological patterns in CRC transcriptomic profiles, enabled accurate tumor–normal classification, and facilitated extraction of biologically meaningful feature importance signals to support high-confidence biomarker discovery.

### Appendix A.2. Computation of Performance Metrics Used in ML Model Evaluation

For the evaluation process, we focused on multiple classification metrics that comprehensively measure different aspects of model performance, including accuracy, precision, recall, F1-score, and AUC-ROC [37,124,125]. The detailed definitions and calculations of these performance metrics are summarized in Table A1, which provides a comprehensive overview of the key evaluation metrics used for distinguishing normal and tumor samples.

**Table A1:** Performance Metrics for the developed Classification Models distinguishing normal and tumor samples.

Name of Metrics	Symbol	Definition
True Positive	(TP)	Correctly predicted the Tumor samples
True Negative	(TN)	Correctly predicted Control samples
False Positive	(FP)	Incorrectly predicted Tumor when it is actually Control
False Negative	(FN)	Incorrectly predicted Control when it is actually Tumor
Recall/Sensitivity/True Positive Rate	(TPR)	$\frac{TP}{TP+FN}$
Accuracy	(ACC)	$\frac{TP+TN}{TP+FP+FN+TN}$
Precision	(PR)	$\frac{TP}{TP+FP}$
(Harmonic mean of Precision and Recall)	F1-Score	$\frac{2\times TP}{2TP+FP+FN}$
Area Under the Curve	(AUC-ROC)	Measures the overall model performance by evaluating TPR vs. FPR across all classification thresholds

Note: True Positive (TP) and True Negative (TN) are correctly classified cases, while False Positive (FP) and False Negative (FN) represent misclassifications. Sensitivity (Recall/TPR) measures the ability to detect tumors, precision (PR) quantifies correct tumor predictions, accuracy (ACC) assesses overall performance, and the F1-score balances precision and recall. The Matthews Correlation Coefficient (MCC) provides a comprehensive evaluation, especially for imbalanced datasets.

### Appendix A.3. Mathematical Formulation of Feature/Gene Selection Methods

To ensure robust, reproducible, and biologically meaningful identification of diagnostic biomarkers, we implemented a multi-strategy feature importance framework that integrates three complementary approaches: tree-based importance derived from ensemble models, SHAP-based model-agnostic interpretation, and coefficient-based importance obtained from regularized linear models. Each approach independently quantifies the contribution of individual genes to model predictions from a distinct methodological perspective. Genes consistently ranked highly across these orthogonal strategies were prioritized as high-confidence candidate biomarkers, forming a consensus-driven selection framework designed to maximize statistical reliability while minimizing method-specific bias.

Tree-based ensemble models were first employed due to their capacity to capture nonlinear relationships, hierarchical decision boundaries, and higher-order gene–gene interactions commonly observed in transcriptomic data [126]. In this framework, feature importance is derived from the contribution of each gene to decision splits across an ensemble of trees. Using the Gini impurity G(t) defined in Equation (A2), the decrease in impurity attributed to a split on gene $f_{j}$ at node t is given by Equation (A13):

(A13) $$\Delta G_{j}(t)=G(t)-(\frac{N_{L}}{N_{t}}G(t_{L})+\frac{N_{R}}{N_{t}}G(t_{R})),$$

with $N_{L}$, $N_{R}$, and $N_{t}$ denoting the number of samples in the left child, right child, and parent node. For an ensemble of M trees, the aggregated importance for gene j is then defined as in Equation (A14)

(A14) $${FI}_{j}^{Tree}=\frac{1}{M}\sum_{m=1}^{M}\sum_{t\in T_{m}:f_{j}=\mathrm{split}(t)}\Delta G_{j}(t)$$

where $T_{m}$ denotes the set of internal nodes in tree m. This formulation was applied to both Random Forest (RF) and Extra Trees (ET). RF uses bootstrapped samples and optimized split thresholds to balance bias and variance, whereas ET introduces additional randomization by selecting split thresholds uniformly at random, improving variance reduction and reducing sensitivity to feature scale.

Complementing these two bagging models, we employed boosting approaches—including Gradient Boosting, XGBoost, and LightGBM—which refine predictions through an iterative correction of residual errors. In these algorithms, feature importance is computed using the gain metric, reflecting the improvement in the objective function (e.g., log-loss) attributable to a split involving a particular gene. For a split at the node t, the gain is calculated as in Equation (A8).

Gain reflects how each gene improves the model’s ability to reduce prediction error across the boosting sequence. Aggregating gain scores across all trees and all boosting iterations allowed us to identify genes that consistently contributed to meaningful improvements throughout model training. All tree-based importance scores were calculated under a 10-fold stratified cross-validation scheme, generating fold-specific importance vectors that were averaged to obtain stable and unbiased estimates.

Because impurity- and gain-based metrics may sometimes produce biased estimates, especially when genes are correlated or appear in multiple redundant pathways, we incorporated a second method: SHAP (SHapley Additive exPlanations). SHAP provides an axiomatic, model-agnostic decomposition of feature contributions grounded in cooperative game theory [38]. For each gene j and sample i, the SHAP value $\phi_{ij}\;$is computed by evaluating the marginal contribution of that gene to all possible subsets of genes as shown in Equation (A15):

(A15) $$\phi_{ij}=\sum_{S\subseteq F\setminus \{j\}}\frac{|S|!\;(|F|-|S|-1)!}{|F|!}[f(S\cup \{j\})-f(S)],$$

where F is the full set of features. SHAP values satisfy local accuracy, consistency, and additivity, and yield the decomposition in Equation (A16):

(A16) $$f(x_{i})=\phi_{i0}+\sum_{j=1}^{p}\phi_{ij},$$

allowing us to interpret each gene’s role in the prediction for both tumor and normal samples. We used SHAP, an efficient algorithm for tree ensembles, to compute exact SHAP values across all five models (RF, ET, GB, XGBoost, and LightGBM). Global SHAP importance was obtained by averaging the absolute values of $\phi_{ij}$ across all samples as shown in Equation (A17):

(A17) $$FI_{j}^{\mathrm{SHAP}}=\frac{1}{n}\sum_{i=1}^{n}|\phi_{ij}|.$$

This approach captures not only linear contributions but also nonlinear dependencies, synergistic gene-gene interactions, and localized effects that may vary between tumor and normal tissues. By examining SHAP distributions, dependence structures, and interaction values, we identified genes that showed stable predictive behavior across models and folds, further strengthening confidence in their biological relevance.

To provide a complementary linear perspective, feature importance was also estimated using regularized linear models, specifically Logistic Regression and linear Support Vector Machines with L2 regularization. These models provide fully interpretable weight vectors whose magnitudes quantify each gene’s marginal contribution to class separation. In accordance with our implementation, both Logistic Regression and linear SVM were applied with L2 regularization, providing stable coefficient estimates in the high-dimensional setting.

Logistic Regression with L2 regularization models the log-odds of tumor versus normal samples as a linear function of gene expression. Given $X\in R^{n\times p}$ and labels y∈{0,1}, the coefficient vector β is estimated by minimizing the penalized negative log-likelihood in Equation (A10).

The linear Support Vector Machine used in our pipeline also employs L2 regularization, consistent with the SVC (kernel = “linear”) implementation. The optimization problem is defined in Equation (A12). All feature importance was quantified as the absolute magnitude of model coefficients, and estimates were obtained within a cross-validation framework, with fold-specific coefficients aggregated to produce stable importance measures.

Following independent estimation of feature importance, gene rankings derived from the three analytical strategies were integrated into a consensus framework by aggregating ranks across methods. To avoid the arbitrary selection of feature set size, a data-driven Top-N optimization procedure was applied, in which candidate thresholds were evaluated using cross-validated performance metrics to identify an optimal trade-off between feature dimensionality and predictive accuracy. Reduced feature sets corresponding to each threshold were assessed using stratified cross-validation, ensuring selection of a parsimonious subset of genes that preserves diagnostic performance. This formulation ensures that the final set of selected genes reflects consistent importance across fundamentally different modeling paradigms while maintaining statistical robustness and generalizability.

### Appendix A.4. Mathematical Formulation of Protein-Protein Network Modeling

The protein–protein interaction (PPI) network was modeled as an undirected graph G=(V,E), where nodes V represent genes/proteins and edges E denote functional or physical interactions. This graph-based representation enables systematic characterization of network topology and facilitates the identification of key hub genes and bottlenecks among the candidate biomarkers.

To quantify node importance from complementary topological perspectives, five centrality measures were computed for each gene: degree, betweenness, closeness, eigenvector [127], and PageRank centrality. Collectively, these metrics capture local connectivity, global reachability, control of information flow, influence via highly connected neighbors, and random-walk–based importance, respectively. Where appropriate, centrality scores were normalized, and a composite multi-metric assessment was used to prioritize biologically relevant hub genes.

**Centrality Measures: Degree Centrality (Local Connectivity).**

Degree centrality captures the number of direct neighbors of a node and reflects its local interaction density as a master regulator. For a node v, degree centrality is defined as in Equation (A18):

(A18) $$C_{D}(v)=deg(v)=\sum_{u\in V}A_{vu}$$

where $A_{vu}$ denotes the adjacency matrix. Genes with high $C_{D}(v)$ act as local hubs and may coordinate multiple colorectal cancer (CRC)-related pathways through numerous direct interactions.

Betweenness centrality (control of information flow).

Betweenness centrality quantifies the extent to which a node lies on shortest paths between other nodes in the network. For node v, it is defined as in Equation (A19):

(A19) $$C_{B}(v)=\sum_{s\neq v\neq t}\frac{\sigma_{st}(v)}{\sigma_{st}}$$

where $\sigma_{st}$ is the total number of shortest paths between nodes s and t, and $\sigma_{st}(v)$ is the number of those paths that pass through v. Nodes with high $C_{B}\left(v\right)\;$function as bridges controlling communication between distinct functional modules.

Closeness centrality (global reachability).

Closeness centrality measures how efficiently a node can reach all other nodes in the network and is defined as in Equation (A20):

(A20) $$C_{C}(v)=\frac{1}{\sum_{u\neq v}d(v,u)}$$

where $d\left(v,u\right)\;$denotes the length of the shortest path between nodes v and u. Nodes with high $C_{C}(v)$ are globally well-positioned to influence diverse biological processes, as they are, on average, only a few steps away from other genes in the network.

Eigenvector centrality (influence of influential neighbors).

Eigenvector centrality extends degree centrality by weighting connections to highly connected neighbors more strongly. It is defined through the eigenvalue Equation (A21):

(A21) $$C_{E}(v)=\frac{1}{\lambda }\sum_{u\in V}A_{vu}\;C_{E}(u)$$

where λ is the largest eigenvalue of the adjacency matrix A, and $C_{E}(u)$ is the eigenvector score of the neighbor u. Nodes with high $C_{E}(v)$ belong to tightly coordinated, highly influential subnetworks and may participate in core regulatory circuits.

PageRank centrality (random-walk–based importance).

PageRank centrality estimates the steady-state probability of visiting a node during a random walk over the network. For node v, PageRank is given by Equation (A22):

(A22) $$PR(v)=\frac{1-d}{N}+d\sum_{u\in \Gamma (v)}\frac{PR(u)}{deg(u)}$$

where d is the damping factor (set to 0.85), N= ∣V∣ is the total number of nodes, and Γ(v) denotes the set of neighbors of v. Nodes with high PR(v) are repeatedly visited during random walks and thus exert system-wide influence beyond their immediate neighborhood.
