# Supplementary material for: Leveraging Multi-Model Machine Learning Algorithms for Tumor–Normal Classification and Discovery of Biomarkers in Colorectal Cancer Using Multi-Omics Data
Source: Cancers (Basel). 2026 May 7;18(10):1503. doi: 10.3390/cancers18101503 (PMC13204554; doi:10.3390/cancers18101503)
Supplement: Supplementary file 1 [file cancers-18-01503-s001.zip › Supplementary Figures documment SD1.pdf]

Supplementary Figures Document SD1: Kaplan-Meier plots showing additional ML-Identified genes that were predictive of survival outcome

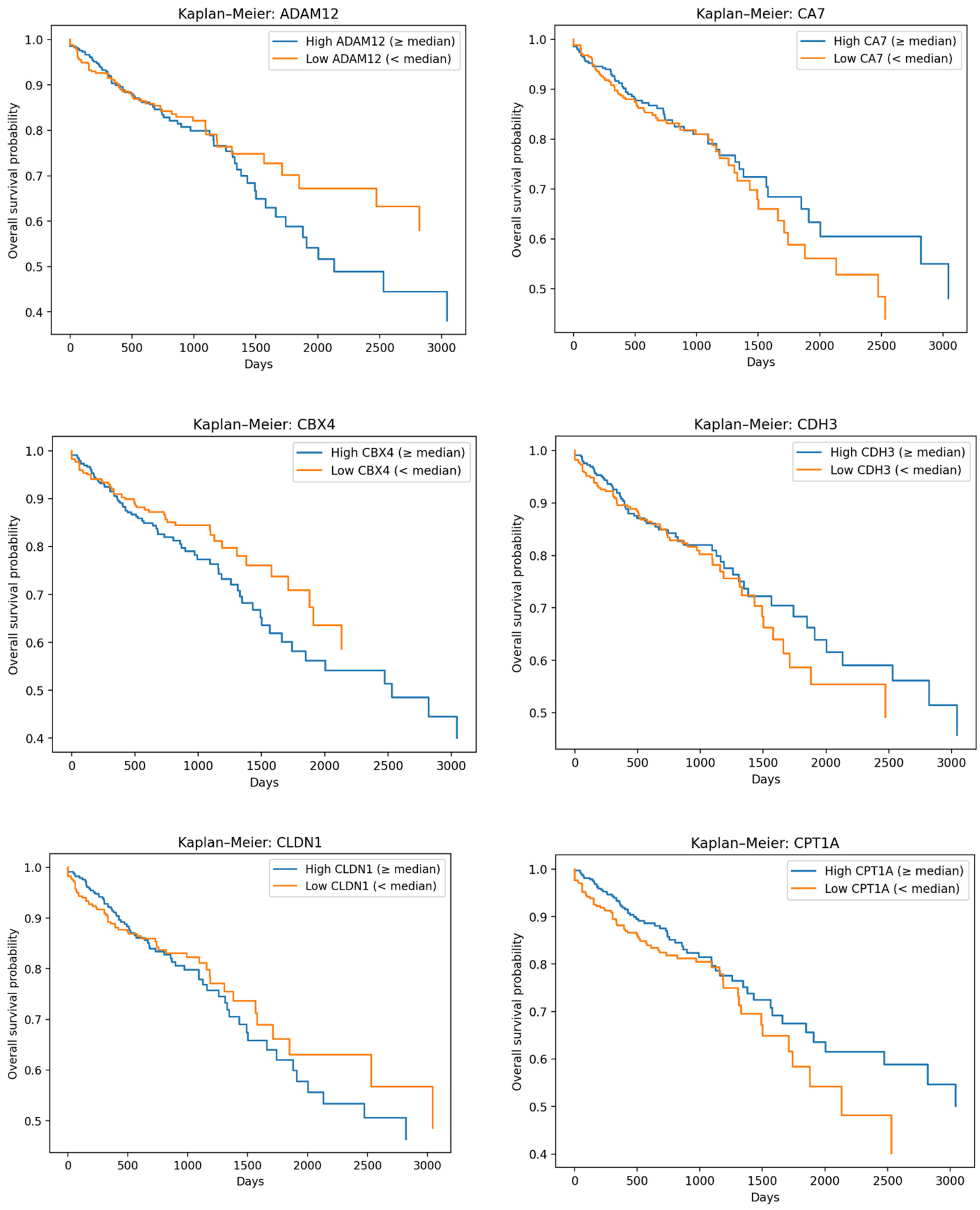

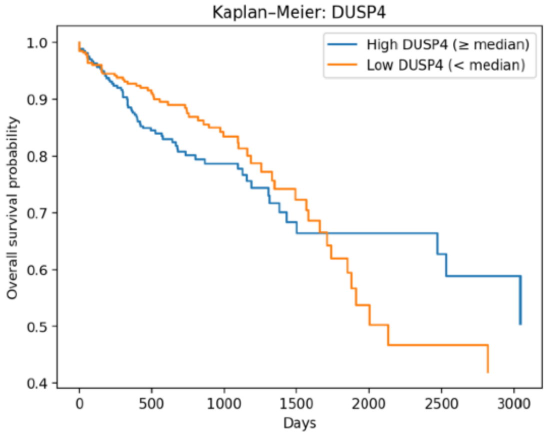
Kaplan–Meier: DUSP4
High DUSP4 (≥ median)
Low DUSP4 (< median)
Overall survival probability
Days

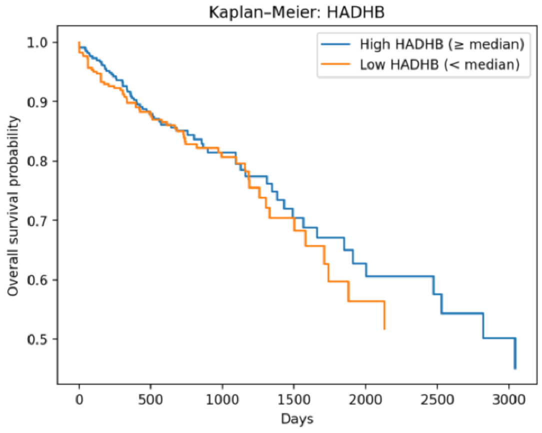
Kaplan–Meier: HADHB
High HADHB (≥ median)
Low HADHB (< median)
Overall survival probability
Days

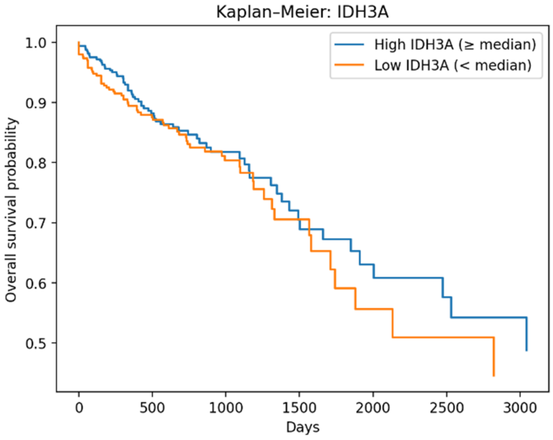
Kaplan–Meier: IDH3A
High IDH3A (≥ median)
Low IDH3A (< median)
Overall survival probability
Days

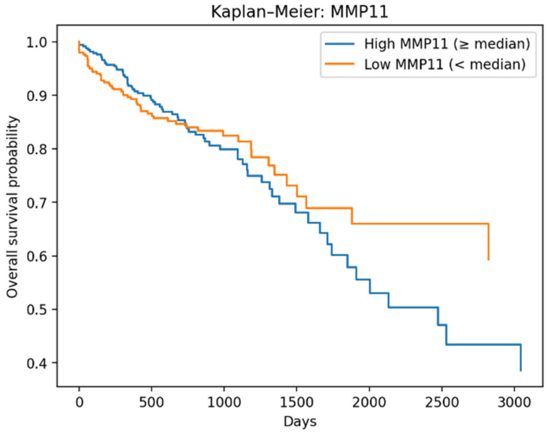
Kaplan–Meier: MMP11
High MMP11 (≥ median)
Low MMP11 (< median)
Overall survival probability
Days

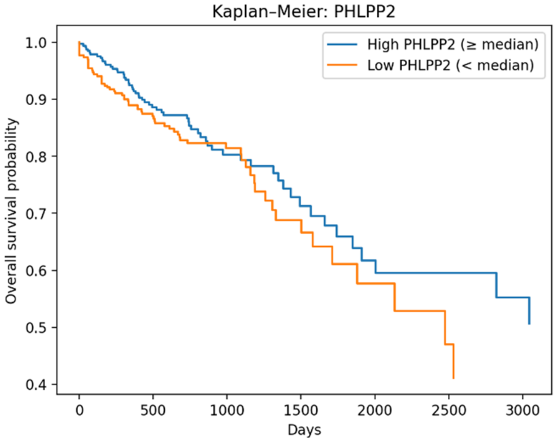
Kaplan–Meier: PHLPP2
High PHLPP2 (≥ median)
Low PHLPP2 (< median)
Overall survival probability
Days

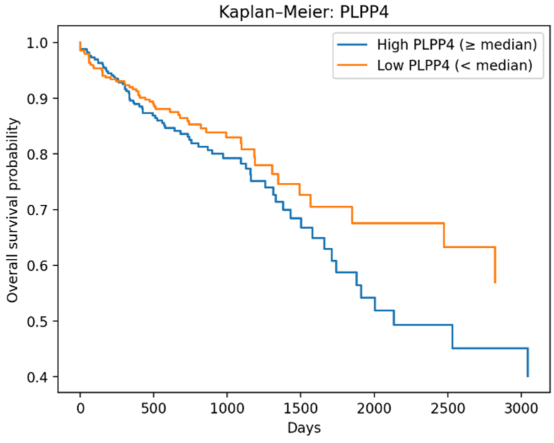
Kaplan–Meier: PLPP4
High PLPP4 (≥ median)
Low PLPP4 (< median)
Overall survival probability
Days

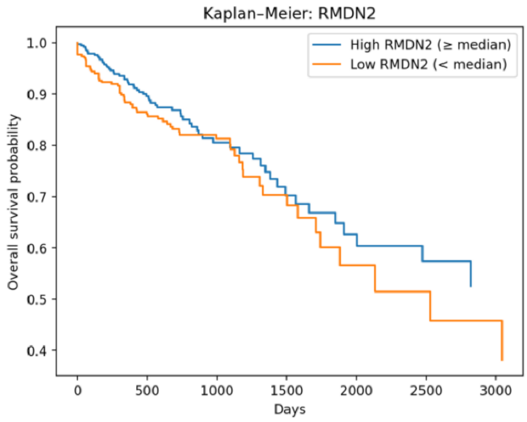
Kaplan–Meier: RMDN2
High RMDN2 (≥ median)
Low RMDN2 (< median)
Overall survival probability
Days

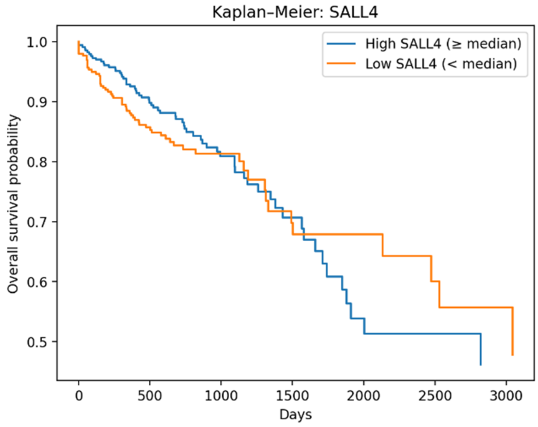
Kaplan–Meier: SALL4
High SALL4 (≥ median)
Low SALL4 (< median)
Overall survival probability
Days

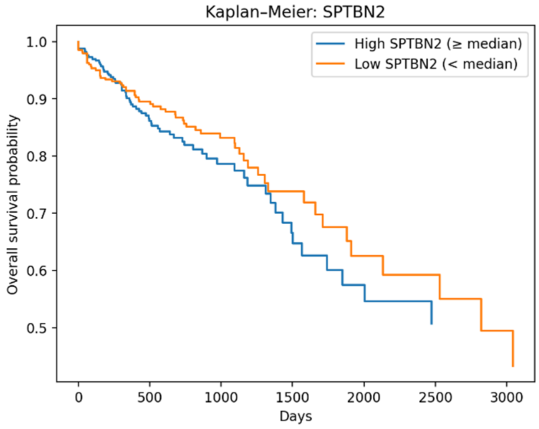
Kaplan–Meier: SPTBN2
High SPTBN2 (≥ median)
Low SPTBN2 (< median)
Overall survival probability
Days

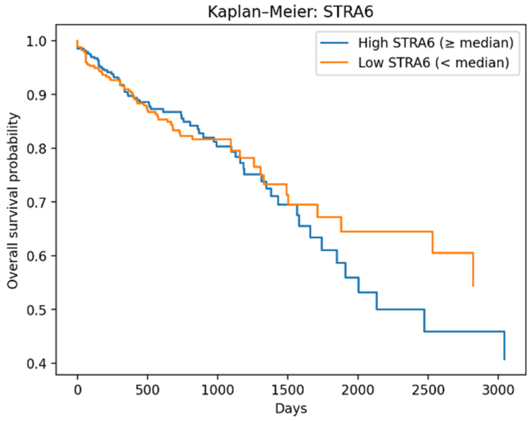
Kaplan–Meier: STRA6
High STRA6 (≥ median)
Low STRA6 (< median)
Overall survival probability
Days

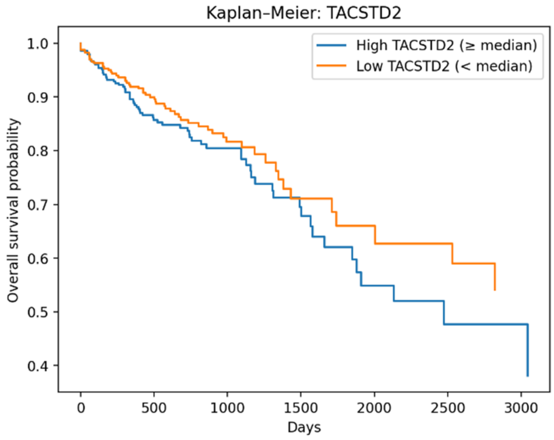
Kaplan–Meier: TACSTD2
High TACSTD2 (≥ median)
Low TACSTD2 (< median)
Overall survival probability
Days

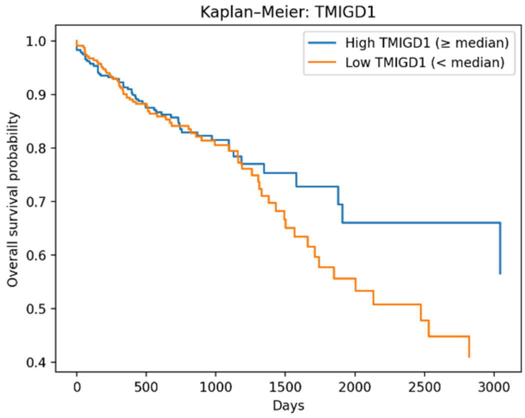
Kaplan–Meier: TMIGD1
High TMIGD1 (≥ median)
Low TMIGD1 (< median)
Overall survival probability
Days

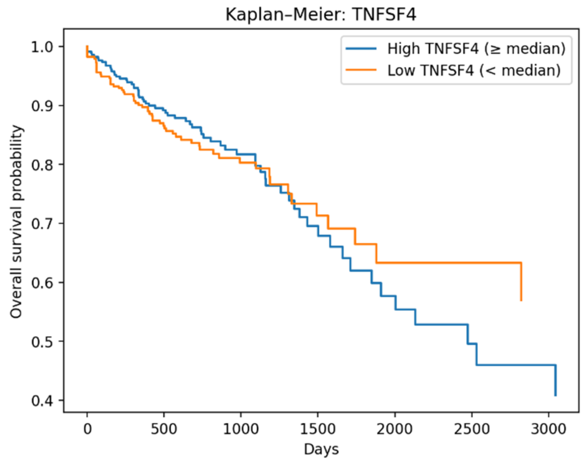
Kaplan–Meier: TNFSF4
High TNFSF4 (≥ median)
Low TNFSF4 (< median)
Overall survival probability
Days
1.0
0.9
0.8
0.7
0.6
0.5
0.4
0
500
1000
1500
2000
2500
3000

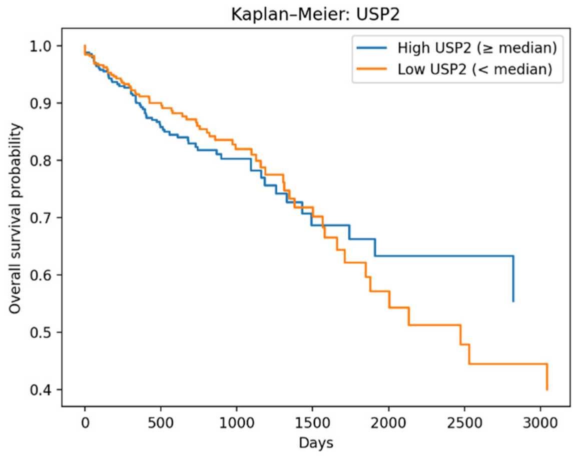
Kaplan–Meier: USP2
High USP2 (≥ median)
Low USP2 (< median)
Overall survival probability
Days
1.0
0.9
0.8
0.7
0.6
0.5
0.4
0
500
1000
1500
2000
2500
3000

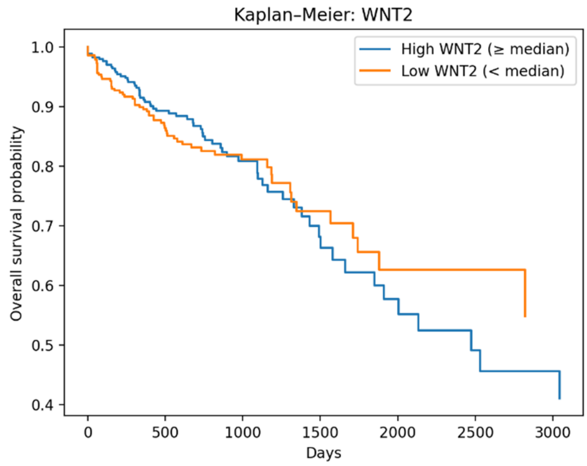
Kaplan–Meier: WNT2
High WNT2 (≥ median)
Low WNT2 (< median)
Overall survival probability
Days
1.0
0.9
0.8
0.7
0.6
0.5
0.4
0
500
1000
1500
2000
2500
3000
