# Supplementary material for: Leveraging Multi-Model Machine Learning Algorithms for Tumor–Normal Classification and Discovery of Biomarkers in Colorectal Cancer Using Multi-Omics Data
Source: Cancers (Basel). 2026 May 7;18(10):1503. doi: 10.3390/cancers18101503 (PMC13204554; doi:10.3390/cancers18101503)
Supplement: Supplementary file 1 [file cancers-18-01503-s001.zip › Supplementary Figure S1.pdf]

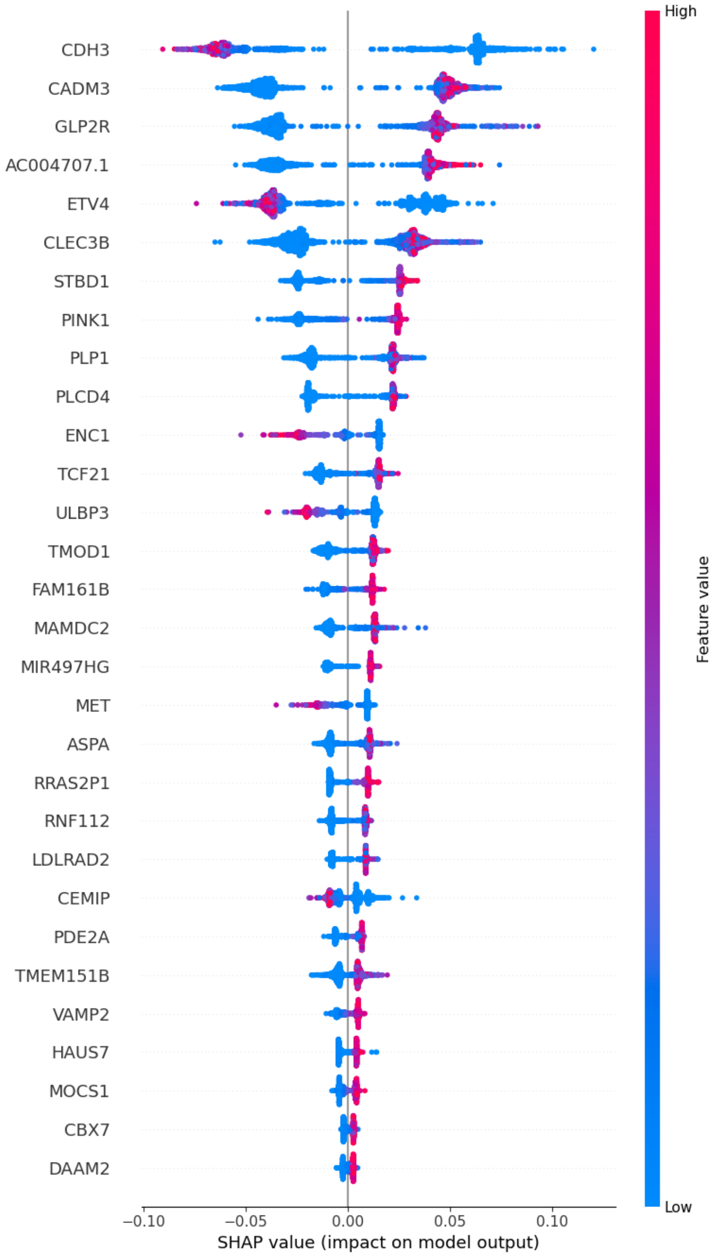

Supplementary Figure S1. SHAP summary plot illustrates the contribution of the top 30 genes to CRC classification.

The plot displays the distribution of SHAP values for each gene across all samples in the Random Forest model. The x-axis represents the SHAP value, indicating both the magnitude and direction of each gene's contribution to model predictions, where positive values shift predictions toward the CRC class and negative values toward the normal class. Each point corresponds to an individual sample, capturing inter-sample variability in feature impact. Color encoding reflects normalized gene expression levels (red = high expression, blue = low expression), enabling direct interpretation of how expression magnitude influences prediction outcomes. Several genes—including *CDH3, GLP2R, CLEC3B, CADM3, ETV4*, and *AC004707.1*—demonstrate strong positive SHAP contributions at higher expression levels, indicating that their upregulation is consistently associated with increased probability of CRC classification. In contrast, genes

such as *STBD1, PINK1, PLCD4, PLP1, ULBP3, TMOD1*, and *MIR497HG* exhibit broader and more symmetric SHAP distributions, suggesting context-dependent effects that may reflect interactions with other molecular features or pathway-level dependencies. Overall, this visualization captures not only the global importance of each gene but also the heterogeneity of their effects across samples, highlighting both dominant drivers of classification and genes with variable, patient-specific contributions—supporting their potential relevance for precision diagnostics.
