# Supplementary material for: Leveraging Multi-Model Machine Learning Algorithms for Tumor–Normal Classification and Discovery of Biomarkers in Colorectal Cancer Using Multi-Omics Data
Source: Cancers (Basel). 2026 May 7;18(10):1503. doi: 10.3390/cancers18101503 (PMC13204554; doi:10.3390/cancers18101503)
Supplement: Supplementary file 1 [file cancers-18-01503-s001.zip › Supplementary Figure S2.pdf]

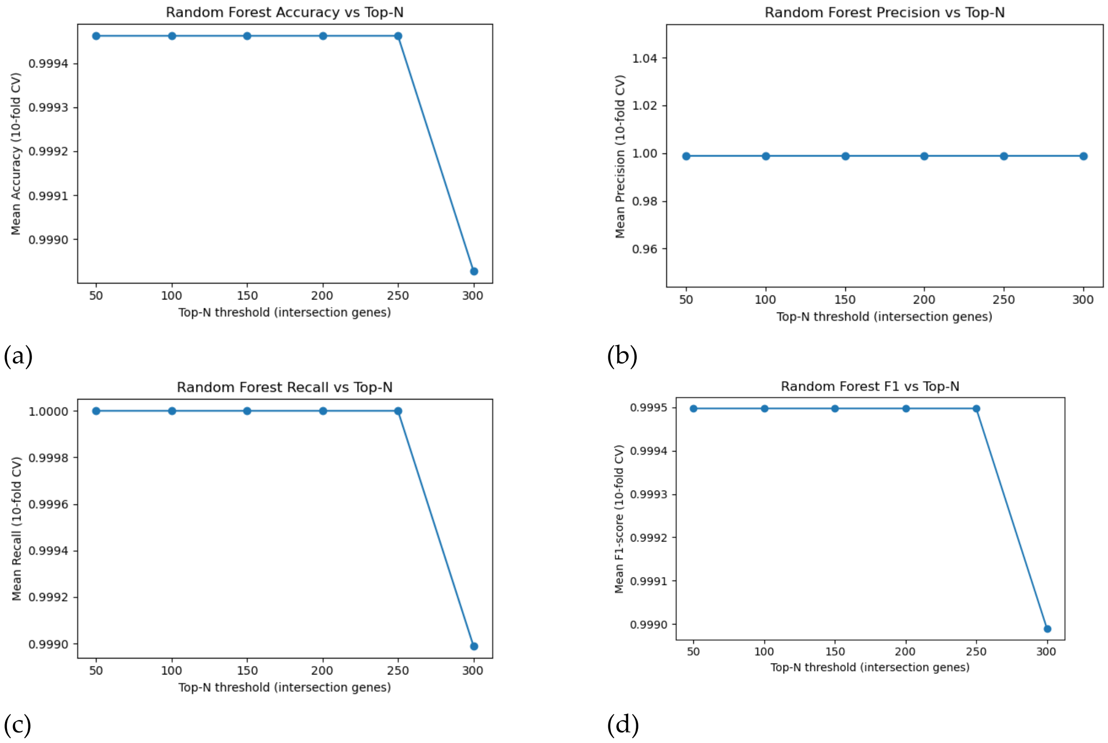

Supplementary Figure S2. Performance of the Random Forest classifier as a function of the Top-N intersection gene threshold. (a) Mean accuracy, (b) mean precision, (c) mean recall, and (d) mean F1-score from stratified 10-fold cross-validation using Top-N meta-ranked genes (N = 50, 100, 150, 200, 250, and 300). Performance remains consistently high for N = 50–250, with a decline at N = 300, indicating that inclusion of lower-ranked genes introduces noise and reduces discriminative power. The optimal threshold of N = 250 was selected as a parsimonious solution, capturing maximal predictive signal without unnecessary feature expansion, as increasing N beyond 250 yields no meaningful performance improvement.
