# Supplementary material for: Leveraging Multi-Model Machine Learning Algorithms for Tumor–Normal Classification and Discovery of Biomarkers in Colorectal Cancer Using Multi-Omics Data
Source: Cancers (Basel). 2026 May 7;18(10):1503. doi: 10.3390/cancers18101503 (PMC13204554; doi:10.3390/cancers18101503)
Supplement: Supplementary file 1 [file cancers-18-01503-s001.zip › Supplementary Figure S3.pdf]

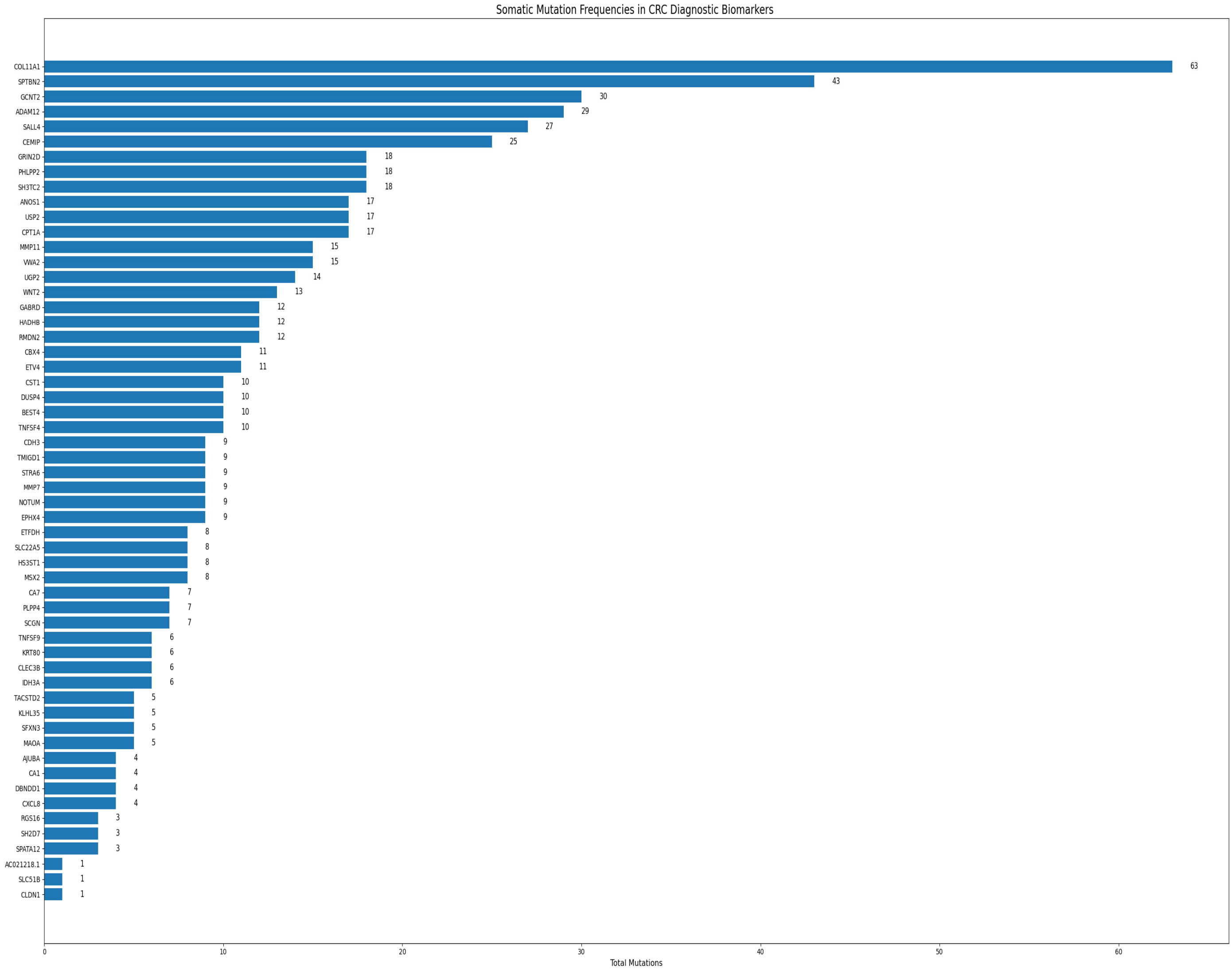

Supplementary Figure S3: Somatic mutation frequencies among the 58-machine learning–derived diagnostic biomarkers using TCGA CRC whole-exome sequencing data. Only genes with at least one mutation are shown. *COL11A1* displayed the highest mutation burden (63 events), followed by *SPTBN2* (43), *GCNT2* (30), *ADAM12 (29), SALL4 (27), and CRIM1 (25*). These recurrent alterations support the biological significance of key genes within the diagnostic signature.
