# Supplementary material for: Leveraging Multi-Model Machine Learning Algorithms for Tumor–Normal Classification and Discovery of Biomarkers in Colorectal Cancer Using Multi-Omics Data
Source: Cancers (Basel). 2026 May 7;18(10):1503. doi: 10.3390/cancers18101503 (PMC13204554; doi:10.3390/cancers18101503)
Supplement: Supplementary file 1 [file cancers-18-01503-s001.zip › Supplementary Figure S4.pdf]

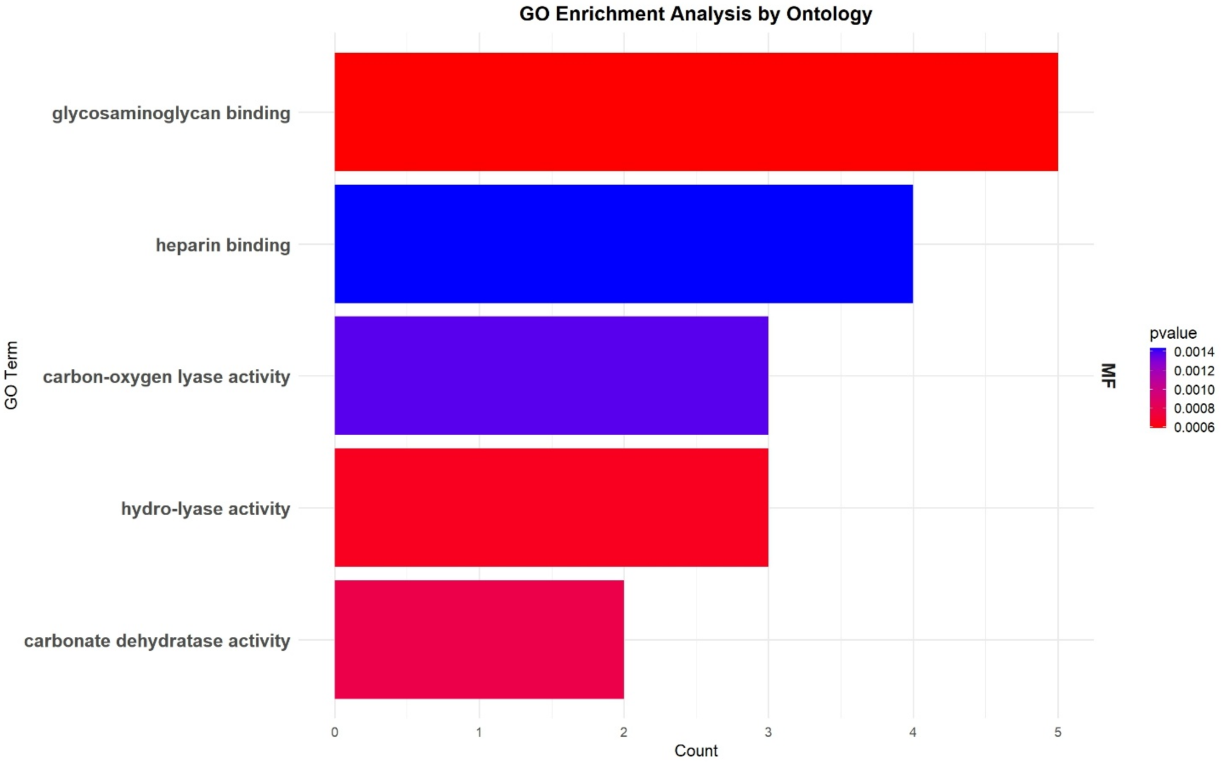

Supplementary Figure S4: Molecular Function (MF) enrichment analysis for the 58 diagnostic biomarker genes. The top enriched MF terms—glycosaminoglycan binding, heparin binding, carbon–oxygen lyase activity, hydro-lyase activity, and carbonate dehydratase activity—are shown with bar lengths indicating gene counts and colors representing adjusted p-values. These enriched biochemical functions highlight key processes involved in colorectal cancer biology, including extracellular matrix remodeling, growth-factor binding, metabolic enzymatic activity, and pH regulation.
